# Satb1 regulates the effector program of encephalitogenic tissue Th17 cells in chronic inflammation

**DOI:** 10.1038/s41467-019-08404-w

**Published:** 2019-02-01

**Authors:** Keiko Yasuda, Yohko Kitagawa, Ryoji Kawakami, Yoshitaka Isaka, Hitomi Watanabe, Gen Kondoh, Terumi Kohwi-Shigematsu, Shimon Sakaguchi, Keiji Hirota

**Affiliations:** 1 Laboratory of Experimental Immunology, Institute for Frontier Life and Medical Sciences, Kyoto University, Kyoto 606-8507, Japan; 2 Department of Experimental Immunology, Immunology Frontier Research Center, Osaka University, Osaka 565-0871, Japan; 3 Department of Nephrology, Osaka University Graduate School of Medicine, Osaka 565-0871, Japan; 4 Laboratory of Integrative Biological Science, Institute for Frontier Life and Medical Sciences, Kyoto University, Kyoto 606-8507, Japan; 5 Department of Orofacial Sciences, University of California, San Francisco, CA 94143 USA

## Abstract

The genome organizer, special AT-rich sequence-binding protein-1 (Satb1), plays a pivotal role in the regulation of global gene networks in a cell type-dependent manner and is indispensable for the development of multiple cell types, including mature CD4^+^ T, CD8^+^ T, and Foxp3^+^ regulatory T cells in the thymus. However, it remains unknown how the differentiation and effector program of the Th subsets in the periphery are regulated by Satb1. Here, we demonstrate that Satb1 differentially regulates gene expression profiles in non-pathogenic and pathogenic Th17 cells and promotes the pathogenic effector program of encephalitogenic Th17 cells by regulating GM-CSF via Bhlhe40 and inhibiting PD-1 expression. However, Satb1 is dispensable for the differentiation and non-pathogenic functions of Th17 cells. These results indicate that Satb1 regulates the specific gene expression and function of effector Th17 cells in tissue inflammation.

## Abstract (web summary)

A chromatin remodelling factor Satb1 is essential for T cell lineage development in the thymus. Here the authors show that while Satb1 is dispensable for the differentiation of Th17 cells and their response to gut commensals, it plays a critical role in pathogenic Th17 effector function in EAE by directly activating Bhlhe40 and modulating PD-1.

## Introduction

Interleukin-17 (IL-17)-producing T-helper 17 (Th17) cells play dichotomous roles in the host defense against pathogens at mucosal surfaces and in the pathogenesis of many inflammatory and autoimmune diseases, such as psoriasis, inflammatory bowel disease, rheumatoid arthritis, and multiple sclerosis^1–7^. Th17 cell differentiation from naive T cells is initiated by transforming growth factor β1 (TGFβ1) and IL-6 and it is further stabilized by environmental cues including cytokines such as IL-1β, IL-23, ligands for the aryl hydrocarbon receptor, hypoxia, and a high sodium chloride concentration^8–16^. Thus, the terminal differentiation and effector functions of Th17 cells are tightly regulated by intrinsic and extrinsic cues in local tissue environments.

Th17 cells exhibit a high degree of functional heterogeneity. The pathogenic effector program of Th17 cells is induced by IL-23 signaling and is characterized by GM-CSF production^17–19^. Induction of Th17 cells by TGF-β1 and IL-6 in vitro is not sufficient to cause autoimmune tissue injury in experimental autoimmune encephalomyelitis (EAE), but when induced by IL-1β, IL-6, and IL-23 or TGF-β3, Th17 cells trigger EAE, consistent with the critical roles of IL-23 signaling in the terminal differentiation of Th17 cells^17, 20–23^. Furthermore, GM-CSF has been identified as a pathogenic signature cytokine of Th17 cells. Driven by IL-1β and IL-23-mediated signaling events along with transcription factor, RORγt, GM-CSF causes local tissue inflammation by recruiting inflammatory myeloid cells^18, 19, 24–26^. Recent transcriptomic studies have attempted to capture the true physiological state of pathogenicity by using ex vivo Th17 cells and identified *Gpr65*, *Toso*, and *Plzp* as novel genes promoting Th17 pathogenicity and CD5 antigen-like (CD5L) as a repressor of Th17 cell-mediated disease^27, 28^. However, apart from the identification of these various determinants of Th17 pathogenicity, a cohesive molecular mechanism that allows for the distinct functioning of pathogenic and non-pathogenic Th17 cells remains to be identified.

Here, we identified special AT-rich binding protein 1 (Satb1), a genome organizer, as a crucial regulator of the pathogenic function of encephalitogenic tissue Th17 cells. We found that Satb1 is dispensable for the differentiation and non-pathogenic function of Th17 cells in the gut but plays a pivotal role in the effector functions of pathogenic Th17 cells, including GM-CSF production via regulation of Bhlhe40 and PD-1 expression in EAE mice. Moreover, gene expression in Th17 cells from the gut and inflamed spinal cord is differentially regulated by Satb1. Thus, our results indicate that inflammatory cues modulate Satb1 to control the specific effector program of tissue Th17 cells.

## Results

### Satb1 is dispensable for non-pathogenic Th17 cells

**Fig. 1:**
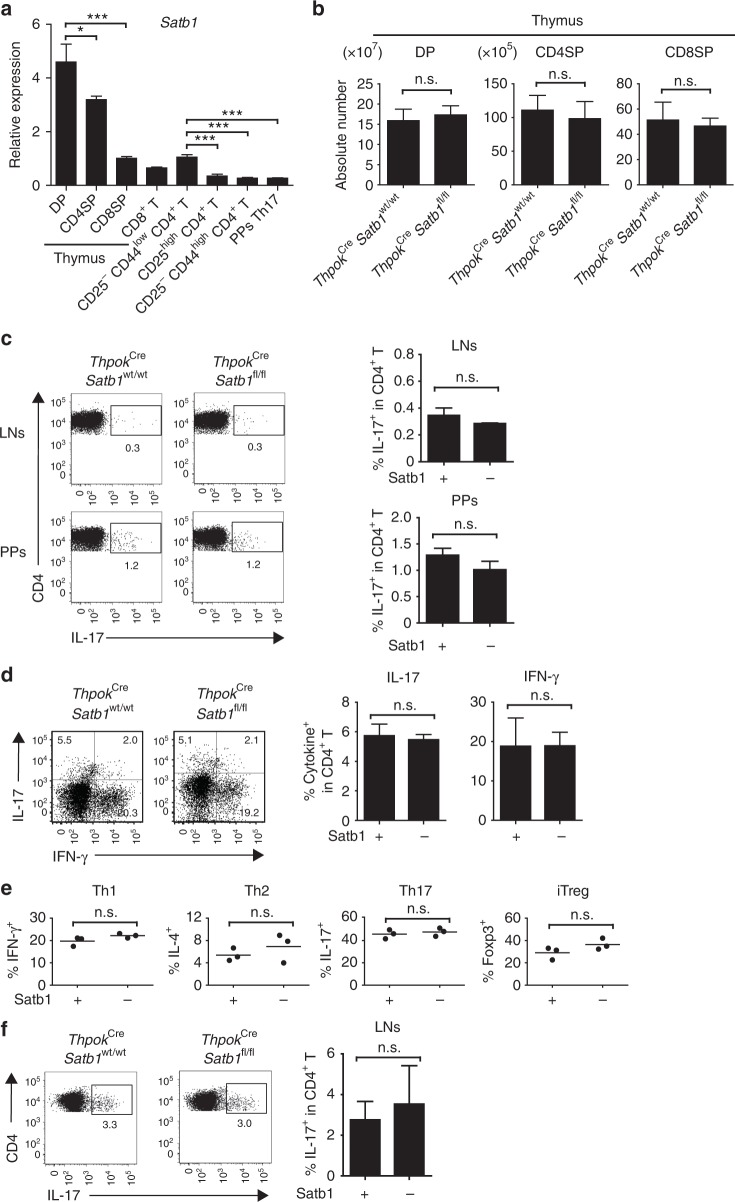
Normal development of non-pathogenic Th17 cells from Satb1-deficient T cells. **a** qPCR of CD4^+^ CD8^+^ (DP), CD4^+^ CD8^−^ (CD4SP), and CD4^−^CD8^+^ (CD8SP) thymocytes and CD8^+^, CD25^−^CD44^low^ CD4^+^, CD25^high^ CD4^+^, CD25^−^ CD44^high^ CD4^+^, and PPs eYFP^+^ CD4^+^ T cells for *Satb1* mRNA expression. **b** Numbers of DP, CD4SP, and CD8SP cells in the thymus of 4-week-old *Thpok*^Cre^ *Satb1*^fl/fl^ and *Thpok*^Cre^ *Satb1*^wt/wt^ littermate controls. **c** Flow cytometry of CD4^+^ T cells from the LNs and PPs of *Thpok*^Cre^ *Satb1*^wt/wt^ and *Thpok*^Cre^ *Satb1*^fl/fl^ mice for intracellular IL-17 expression. The frequencies of IL-17^+^ in CD4^+^ T cells are shown. **d** Flow cytometry of splenic CD4^+^ T cells for intracellular IL-17 and IFN-γ expression. The frequencies of IL-17^+^ and IFN-γ^+^ in CD4^+^ T cells are shown. CD25^−^CD44^low^ CD4^+^ T cells (1 × 10^6^) from *Thpok*^Cre^ *Satb1*^wt/wt^ and *Thpok*^Cre^ *Satb1*^fl/fl^ mice were transferred to *Rag2*^−/−^ mice, and splenic CD4^+^ TCRβ^+^ T cells were analyzed on day 7 after transfer. **e** The frequencies of IFN-γ^+^ Th1, IL-4^+^ Th2, IL-17^+^ Th17 and Foxp3^+^ iTreg cells. CD25^−^CD44^low^ CD4^+^ T cells from *Thpok*^Cre^*Satb1*^wt/wt^ and *Thpok*^Cre^*Satb1*^fl/fl^ mice were cultured under Th1, Th2, Th17, and iTreg conditions for 3 days. Each symbol represents an individual mouse, and the horizontal lines indicate the mean values. **f** Flow cytometry of CD4^+^ T cells from the draining LNs of *Thpok*^Cre^ *Satb1*^wt/wt^ and *Thpok*^Cre^ *Satb1*^fl/fl^ mice immunized with MOG-CFA, staining for CD4 and intracellular IL-17 on day 7 after immunization. The frequencies of IL-17^+^ in CD4^+^ T cells are shown. The bar graphs (**a**–**d**, **f**) show the mean ± s.d. (*n* = 3). The results are representative of three independent experiments (**a–f**). *P < 0.05, ***P < 0.0001 (two-tailed unpaired Student’s *t*-test)

Since Satbl-deficient mice exhibit post-natal lethality^29^, we generated *Thpok*^Cre^ *Satb1*^fl/fl^ mice in which Satbl is conditionally deleted in CD4^+^ CD8^−^ thymocytes and peripheral CD4^+^ T cells^30^. To investigate the differentiation and function of Th17 cells under steady-state conditions, we conducted ex vivo analysis of cells from *Thpok*^Cre^ *Satb1*^wt/wt^ and *Thpok*^Cre^ *Satb1*^fl/fl^ mice. Satb1 was highly expressed in CD4^+^ CD8^+^ thymocytes, as previously reported^31–34^, and the expression was dramatically decreased in peripheral CD8^+^ T and CD4^+^ T cells, including CD25^−^CD44^low^ CD4^+^ naive T, CD25^high^ CD4^+^ regulatory T (Treg), and CD25^−^ CD44^high^ CD4^+^ effector T cells. Satb1 expression was also decreased in Peyer’s patches (PPs) eYFP^+^ CD4^+^ T (Th17) cells purified from an *Il17a*^Cre^ *R26R*^eYFP^ fate reporter strain^35^ (Fig. 1a and Supplementary Fig. 6a and 6c for gating strategies). *Thpok*^Cre^ *Satb1*^fl/fl^ mice had the normal numbers of CD4^+^ CD8^+^, CD4^+^ CD8^−^, and CD4^−^ CD8^+^ thymocytes and the similar frequencies of IL-17-producing CD4^+^ T cells in lymph nodes (LNs) and PPs compared with *Thpok*^Cre^ *Satb1*^wt/wt^ mice (Fig. 1b, c). Upon adoptive transfer into *Rag2*^−/−^ mice, naive T cells from *Thpok*^Cre^ *Satb1*^fl/fl^ mice differentiated into IL-17- or IFN-γ-producing Th cells with the same efficiency as cells from *Thpok*^Cre^ *Satb1*^wt/wt^ mice after homeostatic proliferation (Fig. 1d). By culturing naive CD4^+^ T cells from *Thpok*^Cre^ *Satb1*^fl/fl^ and *Thpok*^Cre^ *Satb1*^wt/wt^ mice, we further examined whether Satb1 was required for the differentiation of Th1, Th2, Th17, and induced Treg (iTreg) subsets in vitro. Although Satb1 expression was relatively increased under Th17 culture conditions (Supplementary Fig. 1), the differentiation of IFN-γ-, IL-4-, IL-17-, and Foxp3-expressing CD4^+^ T cells was not dependent on Satb1 expression in naive T cells (Fig. 1e). Although it was previously reported that the knockdown of Satb1 in human CD4^+^ T cells by siRNA reduced Th2 differentiation^36^, the difference between our result and the report may be attributed to different methods or species we used. We also confirmed that there was the normal differentiation of Th17 cells in *Thpok*^Cre^ *Satb1*^fl/fl^ mice immunized with MOG_35–55_ peptide emulsified in complete Freund’s adjuvant (CFA) (Fig. 1f). Collectively, these results indicate that Satb1 is dispensable for the differentiation of Th17 cells in vivo and for the induction of the Th17 subset in vitro.

### Satb1 regulates the pathogenic function of Th17 cells in EAE

**Fig. 2:**
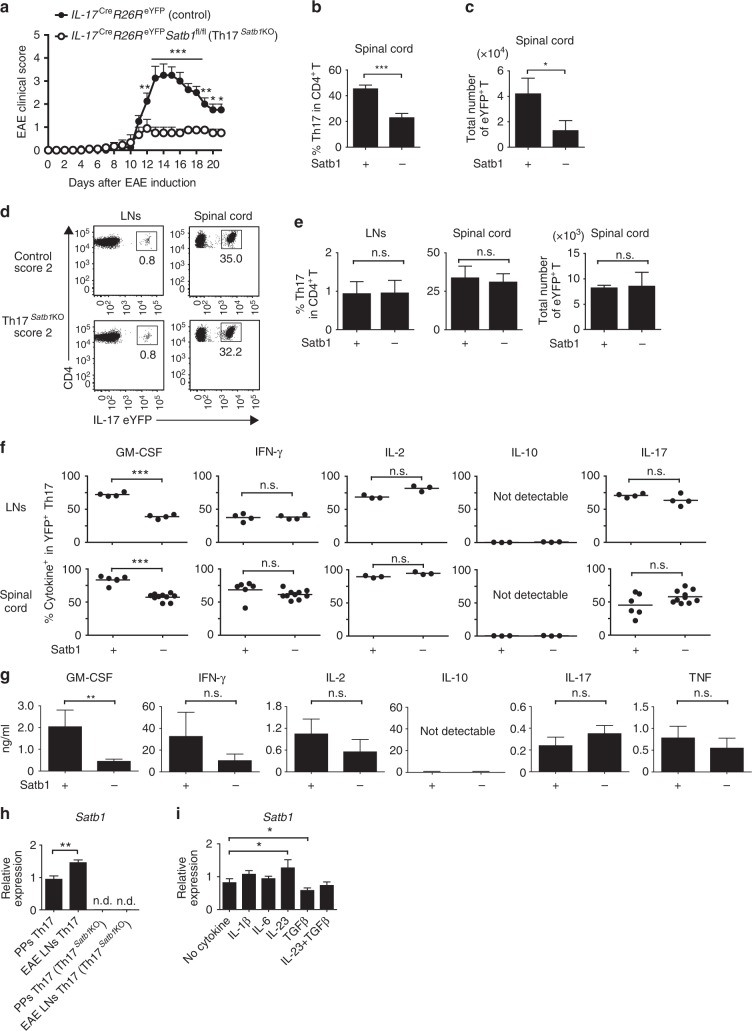
Regulation of pathogenic Th17 functions by Satb1. **a** The mean (+s.e.m.) clinical scores at the days after EAE was induced in *Il17a*^Cre^ *R26R*^eYFP^ *Satb1*^wt/wt^ (control) (*n* = 8) and *Il17a*^Cre^ *R26R*^eYFP^ *Satb1*^fl/fl^ (Th17^*Satb1KO*^) mice (*n* = 7). The incidence of EAE: control 8/8, Th17^*Satb1KO*^ 6/7. *P < 0.05, **P < 0.001, ***P < 0.0001 (two-way ANOVA with Bonferroni’s post-test). **b**, **c** Percentages and absolute numbers of eYFP^+^ Th17 cells in the spinal cord of control and Th17^*Satb1*KO^ mice with mean maximal scores of EAE at the peak of the disease (14 ± 3 days after EAE induction). **d** Flow cytometry of eYFP expression in CD4^+^ T cells from the draining LNs on day 10 and from the spinal cord of the mice showing the same EAE clinical score of 2 at the peak of the disease (on day 17 after EAE induction). **e** The frequencies and the total numbers of eYFP^+^ in CD4^+^ T cells as in (**d**) (*n* = 3). **f** The frequencies of GM-CSF^+^, IFN-γ^+^, IL-2^+^, IL-10^+^, and IL-17^+^ in eYFP^+^ CD4^+^ T cells as in (**d**). Each symbol represents an individual mouse. The horizontal bars indicate the means. **g** Cytokine concentrations in the culture supernatant of re-stimulated Th17 cells are shown. eYFP^+^ Th17 cells were sorted from the spinal cord of control or Th17^*Satb1KO*^ mice at the peak of EAE. Sorted Th17 cells were re-stimulated with plate-coated anti-CD3 for 24 h. **h** qPCR of *Satb1* mRNA expression in eYFP^+^ CD4^+^ T from PPs and draining LNs at day 7 after EAE induction. **i** qPCR of *Satb1* mRNA expression in eYFP^+^ Th17 from the draining LNs of EAE mice on day 7 after re-stimulation with CD3/CD28 Dynabeads in the presence of the indicated cytokines for 24 h. The bar graphs (**b**, **c**, **e**, **g**–**i**) show the mean ± s.d. (*n* = 3). The results are representative of at least three independent experiments (**a**–**i**). *P < 0.05, **P < 0.001, ***P < 0.0001 (two-tailed unpaired Student’s *t*-test)

We next investigated the role of Satb1 in the effector functions of Th17 cells in EAE, an animal model of multiple sclerosis^37, 38^. To determine if Satb1 had a specific role in Th17 cells at the effector phase, we crossed *Satb1*^fl/fl^ mice with *Il17a*^Cre^ *R26R*^eYFP^ mice to generate *Il17a*^Cre^ *R26R*^eYFP^ *Satb1*^fl/fl^ conditional knockout mice, in which Cre-mediated deletion of *Satb1* occurs in Th17 cells upon their differentiation into IL-17-expressing eYFP^+^ CD4^+^ T cells. We refer to these mice as Th17^*Satb1*KO^ henceforth. We immunized them with MOG_35–55_ peptide in CFA to induce EAE^35, 39^. Notably, Th17^*Satb1*KO^ mice were resistant to the development of EAE with fewer eYFP^+^ Th17 cells infiltrating the spinal cord compared with control (*Il17a*^Cre^ *R26R*^eYFP^ *Satb1*^wt/wt^) mice (Fig. 2a–c and Table 1). To examine any defects in the effector functions of Th17 cells lacking Satb1, we analyzed the migration and production of cytokines by Th17 cells from Th17^*Satb1*KO^ and control mice with the same clinical score. Consistent with the normal development of Th17 cells in the absence of Satb1 expression (Fig. 1), the loss of Satb1 did not significantly affect the differentiation or expansion of Th17 cells in the draining LNs and the migration of Th17 cells into the spinal cord with the same EAE clinical score (Fig. 2d, e). Th17 cells lacking Satb1 expressed IFN-γ, IL-2, and IL-17 at levels similar to control cells, but showed impaired production of GM-CSF, a key pathogenic cytokine of the disease^18, 19^ (Fig. 2f). Defective secretion of GM-CSF in Satb1-deficient Th17 cells was confirmed by re-stimulation with an anti-CD3 monoclonal antibody (Fig. 2g and Supplementary Fig. 6b for gating strategies). IL-10, a hallmark of non-pathogenic Th17 cells^17, 40, 41^, was not secreted by control or Satb1-deficient Th17 cells, even upon anti-CD3 re-stimulation (Fig. 2f, g).

**Table 1:** Clinical courses of control and Th17^*Satb1*KO^ mice after EAE induction

	Control (*n* = 8)	Th17^*Satb1*KO^ (*n* = 7)	
Mean maximal score	3.625 ± 0.744	1.000 ± 0.534	*P* < 0.0001
Mean day of onset	11.13 ± 1.885	13.0 ± 2.517	n.s.

The mean (±s.d.) maximal clinical scores and mean (±s.d.) day of onset after EAE was induced in *Il17a*^Cre^ *R26R*^eYFP^ *Satb1*^wt/wt^ (control) (*n* = 8) and *Il17a*^Cre^ *R26R*^eYFP^ *Satb1*^fl/fl^ (Th17^*Satb1*KO^) mice (*n* = 7) as in Fig. 2a

Th17 cells from the draining LNs of control EAE mice had significantly higher levels of Satb1 than those from the PPs (Fig. 2h). In vitro re-stimulation of draining LN Th17 cells from control EAE mice with IL-23, but not IL-1β and IL-6 increased Satb1 expression, whereas TGF-β restrained its effect, consistent with the previous report^42^ (Fig. 2i).

Collectively, these results suggest that Satb1 does not affect the maintenance and migratory capacity of Th17 cells to inflamed tissues, but Satb1 expression is increased upon IL-23 stimulation and plays a pivotal role in the pathogenicity of EAE and effector functions of Th17 cells by regulation of GM-CSF production. IL-1β and IL-6 signaling pathways may interact with Satb1 for the development and function of Th17 cells, but are not involved in the regulation of Satb1.

### Satb1 does not affect activation markers in Th17 cells

**Fig. 3:**
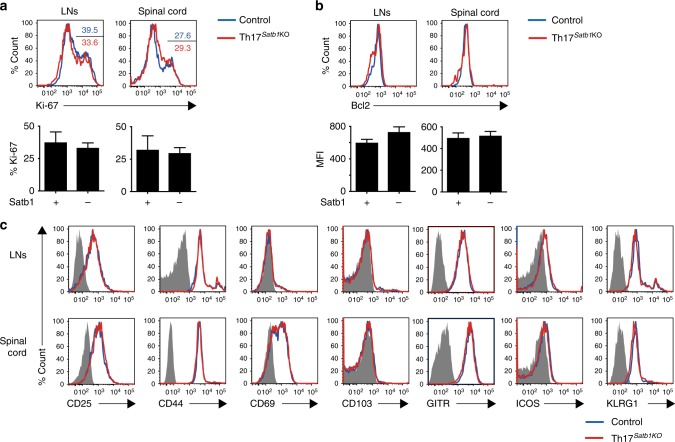
Comparable activation status between control and Satb1-deficient Th17 cells. Flow cytometry of eYFP^+^ CD4^+^ T cells from the draining LNs and the spinal cord for the expression of Ki-67 (**a**), Bcl-2 (**b**), and CD25, CD44, CD69, CD103, GITR, ICOS, and KLRG1 (**c**) as in Fig. 2d. The frequencies of Ki-67^+^ (**a**) and the mean fluorescence intensity (MFI) of Bcl-2^+^ in eYFP^+^ CD4^+^ T cells (**b**) are shown. The bar graphs (**a** and **b**) show the mean ± s.d. (*n* = 3). The results are representative of at least three independent experiments (**a**–**c**)

Given that Satb1 influences the regulation of multiple genes in T cells and their precursors^29, 39, 43^, we compared the activation and proliferation profile of eYFP^+^ Th17 cells from Th17^*Satb1*KO^ and control mice after EAE induction. The expression levels of Ki-67 and Bcl-2 in Th17 cells from draining LNs and the spinal cord were comparable between the two mouse strains (Fig. 3a, b). Further, the absence of Satb1 in effector Th17 cells did not affect the expression of surface markers CD25, CD44, CD69, CD103, GITR, ICOS, and KLRG1 (Fig. 3c), indicating that Satb1 does not regulate these activation-, proliferation-, and survival-associated genes in effector Th17 cells.

### Satb1 regulates pathogenic gene expression in Th17 cells

**Fig. 4:**
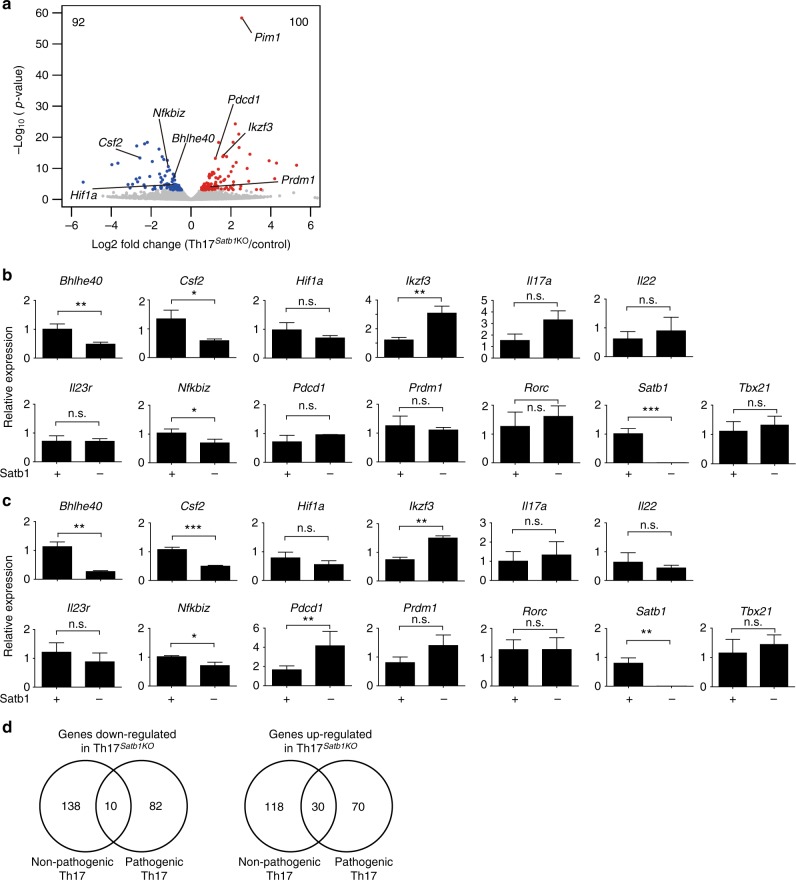
Differential gene expression in pathogenic and non-pathogenic Th17 cells from control and Th17^*Satb1*KO^ mice. **a** RNA-seq analysis of eYFP^+^ CD4^+^ T cells from the spinal cord of control and Th17^*Satb1*KO^ mice after EAE induction. eYFP^+^ Th17 cells were sorted at the peak of the disease (14 days after EAE induction). The volcano plot shows the differential gene expression between the Th17^*Satb1*KO^ versus control eYFP^+^ CD4^+^ T cells. The genes up-regulated in Th17^*Satb1*KO^ eYFP^+^ CD4^+^ T cells are shown in red, and the genes down-regulated are shown in blue. Two biological replicates were analyzed. **b**, **c** qPCR of eYFP^+^ CD4^+^ T cells from the draining LNs (**b**) and spinal cord (**c**) as in (**a**) for the expression of *Bhlhe40*, *Csf2*, *Hif1a*, *Ikzf3*, *Il17a*, *Il22*, *Il23r*, *Nfkbiz*, *Pdcd1*, *Prdm1*, *Rorc*, *Satb1*, and *Tbx21*. eYFP^+^ Th17 cells from the draining LNs were sorted on days 7–14 after EAE induction, and eYFP^+^ Th17 cells from spinal cord was sorted at the peak of the disease (14 ± 3 days after EAE induction). **d** Venn diagram presenting overlap of the number of down-regulated or up-regulated genes between PP eYFP^+^ CD4^+^ T cells (non-pathogenic) and eYFP^+^ CD4^+^ T cells (pathogenic) from the inflamed spinal cord of Th17^*Satb1*KO^ mice after EAE induction. eYFP^+^ Th17 cells were sorted at the peak of the disease (14 days after EAE induction). The bar graphs (**b** and **c**) show the mean ± s.d. (*n* = 3). *P < 0.05, **P < 0.001, ***P < 0.0001 (two-tailed unpaired Student’s *t*-test)

Since Satb1-deficient Th17 cells exhibited no changes in the expression of key activation and proliferation markers (Fig. 3), global expression profiling was conducted to identify other dysregulated genes associated with the loss of Satb1. To this end, eYFP^+^ Th17 cells were sorted from the spinal cords of Th17^*Satb1*KO^ and control mice at the peak of the disease after EAE induction and were subjected to gene expression analysis by RNA-seq. As shown in Fig. 3, Satb1 deficiency showed limited effect on global gene expression. We identified 192 differentially expressed genes, with 92 down-regulated and 100 up-regulated genes, in Satb1-deficient Th17 cells compared with control Th17 cells (Fig. 4a). Among these, we validated *Satb1* and 12 other potential candidates associated with Th17 pathogenicity by q-PCR (Fig. 4b, c). Of the 12 genes, 3 genes (*Bhlhe40, Csf2*, and *Nfkbiz*) were significantly down-regulated in Satb1-deficient Th17 cells from both the draining LNs and spinal cord, 1 gene (*Ikzf3*) was significantly up-regulated in Satb1-deficient Th17 cells from both the draining LNs and spinal cord, and 1 gene (*Pdcd1*) was significantly up-regulated in Satb1-deficient Th17 cells from only the spinal cord (Fig. 4c). *Csf2* encodes GM-CSF and *Bhlhe40* encodes a key transcription factor driving *Csf2* transcription^44, 45^; therefore, their down-regulation is consistent with the impaired production of GM-CSF by Satb1-deficient Th17 cells (Fig. 2f, g). *Nfkbiz* encodes a transcriptional coregulator that acts with RORγt to regulate IL-17 expression in Th17 cells^46^; the effect was likely to be limited because of the normal development of Th17 cells and IL-17 production in the absence of Satb1. By contrast, the expression of *Ikzf3*, which promotes the development of Th17 cells by repressing IL-2 production^47^, was significantly higher in Satb1-deficient Th17 cells despite their ability to secrete IL-2 (Fig. 2f). There was no significant difference in the expression of genes encoding Th17 signature cytokines and regulators such as *Hif1a*, *Il17a*, *Il22*, *Il23r*, *Prdm1*, and *Rorc* confirmed by q-PCR (Fig. 4b, c). Notably, the differential regulation of *Bhlhe40* and *Pdcd1*, which encodes the inhibitory receptor PD-1, was not seen in non-pathogenic Th17 cells in PPs, which barely secreted GM-CSF (Supplementary Fig. 2a-c). Gene expression analysis revealed that the majority of genes dysregulated in the absence of Satb1 was not overlapped between non-pathogenic and pathogenic Th17 cells. ~150 genes were up-regulated and down-regulated in non-pathogenic Satb1-deficient Th17 cells, but did not affect the signature cytokine *Il17* and *Il22* expression and transcription factors including *Bhlhe40* (Fig. 4d and Supplementary Fig. 2d). The specific role of the overlapped genes (10 down-regulated and 30 up-regulated) in other immune reactions remains to be defined in the future study (Table 2). Taken together, these results indicate that Satb1 specifically regulates the divergent gene expression in non-pathogenic and pathogenic Th17 cells at different locations under homeostatic and inflammatory conditions.

**Table 2:** The list of the overlapped genes shown in Fig. 4d

10 Down-regulated genes	30 Up-regulated genes
5430421N21Rik	A930015D03Rik
Ccr7	Aplp2
Cd9	C920025E04Rik
Galnt9	Cd226
Lrig1	Ddx28
Mocs1	Dkk3
St8sia1	F2r
Tbc1d4	Glo1
Tgfbr3	Gpx1
Tnfsf11	Gzma
	H2-T10
	H2-T24
	Hlf
	Ifi203
	Il10ra
	Lrrn3
	Pim1
	Plek
	Plekhf1
	Plxdc2
	Ppp1r14c
	Prdm1
	Rbm24
	Sept11
	Slc25a24
	Stom
	Tns4
	Tubb5
	Uhrf2
	Zfand6

The list of the overlapped genes down-regulated or up-regulated between non-pathogenic (PP eYFP^+^ CD4^+^) Th17 cells and pathogenic (eYFP^+^ CD4^+^) Th17 cells from the inflamed spinal cord of Th17^*Satb1*KO^ mice after EAE induction. eYFP^+^ Th17 cells were sorted at the peak of the disease (14 days after EAE induction)

### Bhlhe40 restores the pathogenicity of Satb1^−/−^ Th17 cells

**Fig. 5:**
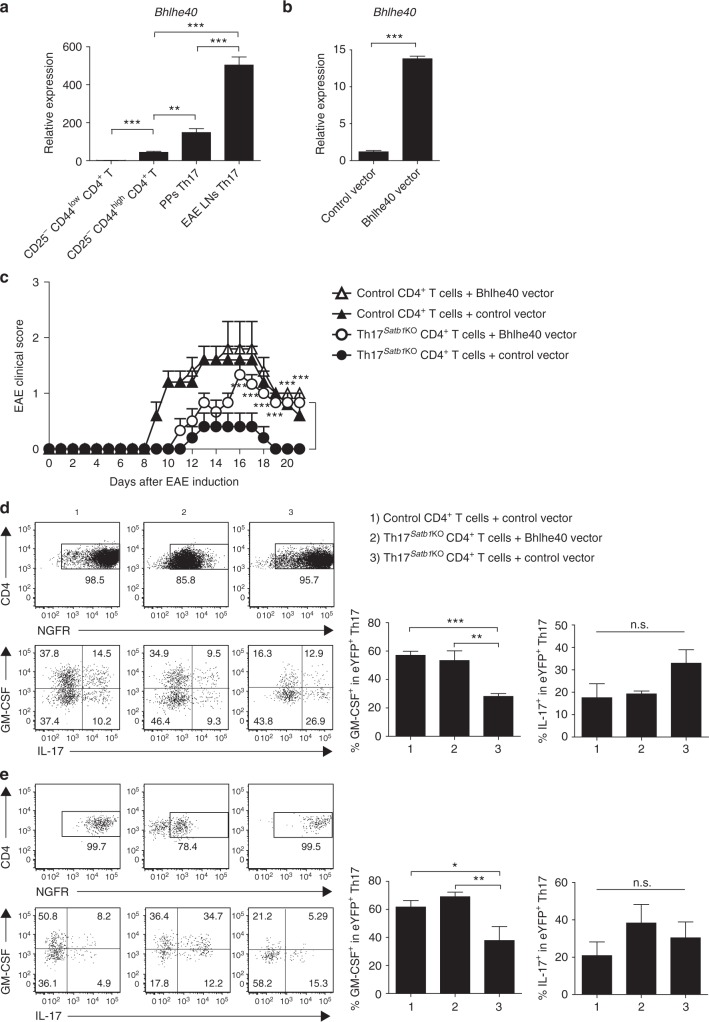
Retroviral transduction of Bhlhe40 restores the pathogenic function of Satb1-deficient Th17 cells. **a** qPCR of *Bhlhe40* mRNA expression in CD25^−^ CD44^low^ CD4^+^, CD25^−^ CD44^high^ CD4^+^, PPs eYFP^+^ CD4^+^, and EAE LNs eYFP^+^ CD4^+^ T cells. EAE LN eYFP^+^ Th17 cells were sorted on days 7–14 after EAE induction. **b** qPCR of *Bhlhe40* mRNA expression 3 days after Bhlhe40 was retrovirally transduced in naive CD25^−^CD44^low^ CD4^+^ T cells. **c** Mean (+s.e.m.) clinical scores on the days after EAE induction in *Rag2*^−/−^ mice; the following conditions were followed for the transfer of NGFR^+^ CD4^+^ T cells (2 × 10^6^): (1) control CD4^+^ T cells transduced with the Bhlhe40 vector (*n* = 5), (2) control CD4^+^ T cells transduced with the control vector (*n* = 5), (3) Th17^*Satb1*KO^ CD4^+^ T cells transduced with the Bhlhe40 vector (*n* = 6), and (4) Th17^*Satb1*KO^ CD4^+^ T cells transduced with the control vector (*n* = 5). The incidence of EAE was as follows: (1) 5/5, (2) 5/5, (3) 6/6, and (4) 2/5. ***P < 0.0001 (two-way ANOVA with Bonferroni’s post-test). **d**, **e** Flow cytometry of CD4^+^ T cells (top) and NGFR^+^ eYFP^+^ CD4^+^ T cells (bottom) in draining LNs (**d**) and spinal cord (**e**) as in (**c**) for IL-17 and GM-CSF expression on day 14 after EAE inductions. The frequencies of GM-CSF^+^ in eYFP^+^ CD4^+^ T cells are shown. The bar graphs (**a**, **b**, **d**, **e**) show the mean ± s.d. (*n* = 3). The results are representative of two independent experiments. **P < 0.001; ***P < 0.0001 (two-tailed unpaired Student’s *t*-test or one-way ANOVA)

We next focused on Bhlhe40 as a possible regulatory factor of Satb1-mediated GM-CSF production. The expression of *Bhlhe40* was higher in pathogenic Th17 cells from the draining LNs of control EAE mice than in non-pathogenic Th17 cells from PPs and CD25^−^CD44^high^ CD4^+^ effector T cells (Fig. 5a), correlating with the increased expression of Satb1 in Th17 cells from the draining LNs of control EAE mice (Fig. 2h). To examine the function of *Bhlhe40* in Satb1-deficient Th17 cells in which *Bhlhe40* expression was decreased, we constructed a retroviral vector to ectopically express Bhlhe40 and NGFR as a reporter gene and validated the induction of *Bhlhe40* in NGFR^+^ T cells after Bhlhe40 was transduced in naive CD4^+^ T cells under Th0 conditions (Fig. 5b). We then addressed whether the Satb1–Bhlhe40 axis plays a critical role in the pathogenesis of Th17 cells. Retroviral transduction of a Bhlhe40-expressing vector into Satb1-deficient CD4^+^ T cells, followed by adoptive transfer into *Rag2*^−/−^ mice in which EAE was induced 2 weeks later, substantially restored the pathogenic function of Satb1-deficient CD4^+^ T cells (Fig. 5c and Supplementary Fig. 6d for gating strategies), as well as GM-CSF expression in the draining LNs (Fig. 5d) and spinal cord (Fig. 5e) of EAE recipient mice. Collectively, these results indicate that Satb1 regulates the pathogenic function of Th17 cells and GM-CSF production in part via Bhlhe40.

### Satb1 inhibits PD-1 expression in effector Th17 cells

**Fig. 6:**
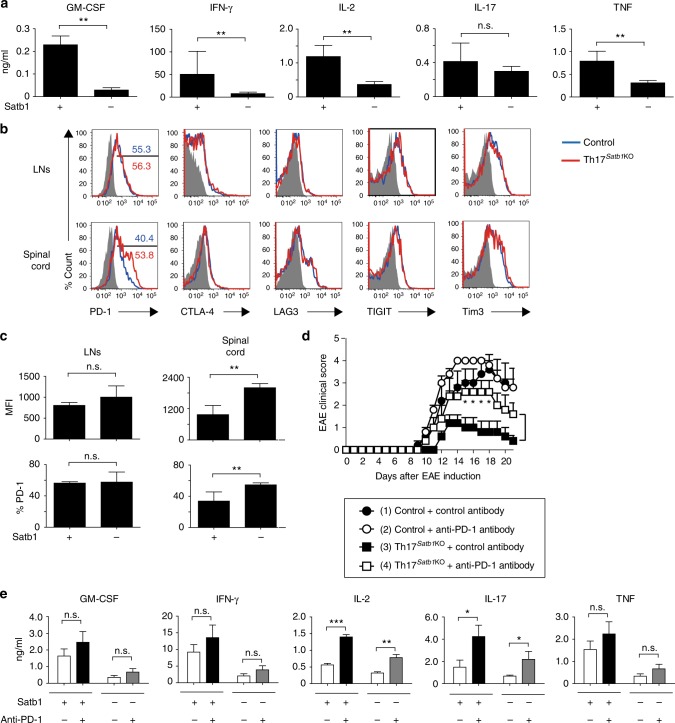
Satb1 inhibits PD-1 expression by Th17 cells in the spinal cord. **a** Cytokine concentrations in the culture supernatant of re-stimulated Th17 cells are shown. eYFP^+^ Th17 cells were sorted from the spinal cord of control or Th17^*Satb1KO*^ mice at the peak of EAE. Sorted Th17 cells were re-stimulated with BMDCs in the presence of MOG_35–55_ peptide (50 μg/ml) for 24 h. **b**, **c** Flow cytometry of draining LNs (day 7 after EAE induction) and spinal cord (day 14 ± 3 after EAE induction) eYFP^+^ CD4^+^ T cells as in Fig. 2d for the expression of PD-1, CTLA-4, LAG3, TIGIT, and Tim3. The frequencies and mean fluorescence intensity (MFI) of PD-1^+^ in eYFP^+^ CD4^+^ T cells are shown (**c**). **d** The mean (+s.e.m.) clinical scores on the days after EAE was induced in control or Th17^*Satb1*KO^ mice, which were treated with the isotype-matched control (IgG2a) or anti-PD-1 mAbs (*n* = 5). The incidence of EAE was as follows: (1) 5/5, (2) 5/5, (3) 4/5, and (4) 5/5. *P < 0.05 (two-way ANOVA with Bonferroni’s post-test). **e** Cytokine concentrations in the culture supernatant of re-stimulated Th17 cells are shown. eYFP^+^ Th17 cells were sorted from the spinal cord of control or Th17^*Satb1KO*^ mice at the peak of EAE and then re-stimulated with BMDCs in the presence of MOG_35–55_ peptide (50 μg/ml) with or without the PD-1 antibody (20 μg/ml) for 24 h. The bar graphs (**a**, **c**, **e**) show the mean ± s.d. (*n* = 3). The results are representative of three independent experiments. *P < 0.05; **P < 0.001; ***P < 0.0001 (two-tailed unpaired Student’s *t*-test)

In an attempt to characterize the impaired effector functions of Th17 cells in the absence of Satb1, we found that the production of GM-CSF, IFN-γ, IL-2, and TNF, but not IL-17, was greatly impaired when Satb1-deficient Th17 cells from the inflamed spinal cord of Th17^*Satb1*KO^ mice were cultured with bone marrow-derived dendritic cells (BMDCs) in the presence of MOG_35–55_ peptide while GM-CSF and IL-2 were also affected in Satb1-deficient Th17 cells from the spleen (Fig. 6a and Supplementary Fig. 3a). Since the production of IFN-γ, IL-2, and TNF upon re-stimulation with anti-CD3 was comparable between Satb1-deficient and control Th17 cells (Fig. 2g), and the communication between dendritic cells and T cells is based on receptor–ligand interactions that regulate immune checkpoints, BMDC-mediated suppression of effector cytokines prompted us to investigate the expression of immune checkpoint molecules by Th17 cells^48^. Satb1-deficient Th17 cells from the inflamed spinal cord, but not the draining LNs and spleen of EAE mice, expressed higher levels of PD-1 than control Th17 cells in accordance with the up-regulation of *Pdcd-1* observed in Satb1-deficient Th17 cells and the similar finding reported in activated T cells^42^ (Fig. 4b, c and 6b, c and Supplementary Fig. 3b). The expression of other immune checkpoint molecules, such as CTLA-4, LAG3, TIGIT, and Tim3, was unchanged in Satb1-deficient Th17 cells from the spinal cord, draining LNs and spleen (Fig. 6b and Supplementary Fig. 3b).

We next examined whether the up-regulation of PD-1 by Satb1-deficient Th17 cells is related to the decreased severity of EAE seen in Th17^*Satb1*KO^ mice (Fig. 2a). To this end, anti-PD-1 blocking monoclonal antibody was administered to mice following EAE induction. Th17^*Satb1*KO^ mice that received the anti-PD-1 antibody exhibited increased severity of disease compared to Th17^*Satb1*KO^ mice that received the isotype-matched control antibody. Disease severity in control mice was comparable between anti-PD-1 and control antibody-treated mice (Fig. 6d). Moreover, when Th17 cells from control or Th17^*Satb1*KO^ inflamed spinal cords were co-cultured with BMDCs in the presence of MOG_35–55_ peptide and the anti-PD-1 antibody, the production of IL-2 and IL-17 by control or Satb1-deficient Th17 cells was significantly enhanced, whereas the production of GM-CSF, IFN-γ, IL-1β, IL-6, IL-23, and TNF was unaffected by the presence of the anti-PD-1 antibody (Fig. 6e and Supplementary Fig. 3c), indicating that PD-1 signaling predominantly regulates IL-2 and IL-17 production, but not all the effector cytokines including GM-CSF, which appears to be regulated by Bhlhe40 in Th17 cells. Taken together, these results indicate that Satb1-mediated regulation of PD-1 expression controls specific effector cytokines including IL-17 by Th17 cells and contributes to the pathogenesis of EAE.

### Satb1 binds to the promoter region at the *Bhlhe40* locus

**Fig. 7:**
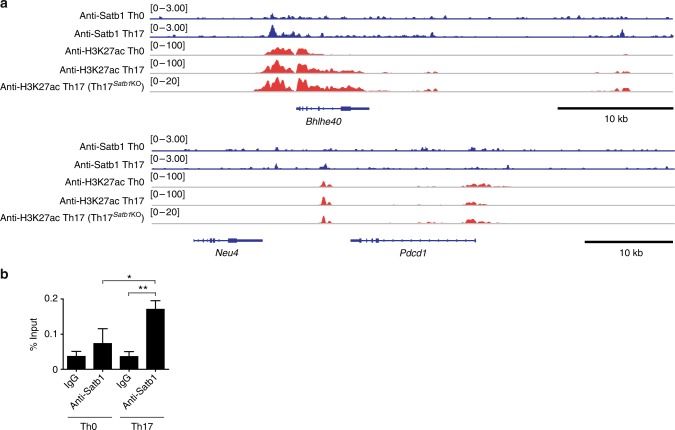
Direct binding of Satb1 at the *Bhlhe40* active promoter region. **a** ChIP-seq analyses of Satb1 binding and H3K27ac modification at the *Bhlhe40* and *Pdcd1* loci in in vitro polarized Th0 or eYFP^+^ Th17 cells. **b** Quantitative ChIP-PCR analysis for Satb1 binding to the *Bhlhe40* promoter region. Chromatin from in vitro polarized Th0 or eYFP^+^ Th17 cells was immune-precipitated with anti-Satb1 antibody or control IgG. Percent of input before IP (1% gel input values) was quantified by real-time qPCR using primers specific for Bhlhe40 promoter region. The bar graph shows the mean ± s.d. (*n* = 3). The results are representative of more than two independent experiments. *P < 0.05; **P < 0.001 (two-tailed unpaired Student’s *t*-test)

To gain further insight into Satb1-dependent transcriptional regulation in Th17 cells, we investigated genome-wide Satb1-binding sites in Th17 cells using chromatin immunoprecipitation sequencing (ChIP-seq) analyses with an anti-Satb1 monoclonal antibody. In vitro polarized Satb1-deficient eYFP^+^ Th17 cells showed higher levels of PD-1 than control eYFP^+^ Th17 cells (Supplementary Fig. 4a), consistent with increased PD-1 expression observed in Th17 cells from the inflamed spinal cords (Fig. 6b). Using eYFP^+^ Th17 cells from *Il17a*^Cre^ *R26R*^eYFP^ *Satb1*^wt/wt^ mice, we analyzed the Satb1 binding sites at affected gene loci in Th17 cells (loci identified in Fig. 4a). Among the gene loci analyzed (*Bhlhe40*, *Pdcd1*, *Csf2*, *Hif1a*, *Ikzf3*, *Nfkbiz*, and *Prdm1*), Satb1 specifically bound only to the active promoter region of the *Bhlhe40* locus, which was marked by H3K27 acetylation and this association of Satb1 with the enhancer region was also confirmed by quantitative ChIP-PCR (Fig. 7a, b and Supplementary Fig. 4b). However, this active enhancer mark of H3K27ac in Th17 cells could be maintained independently of Satb1. Notably, there was no direct evidence of Satb1 binding on the *Pdcd1*, *Csf2*, *Hif1a*, *Ikzf3*, *Nfkbiz*, and *Prdm1* loci. These results suggest that there are direct and context-dependent gene regulations by Satb1 that control GM-CSF and PD-1 expression in encephalitogenic tissue Th17 cells, but Satb1 is not involved in the epigenetic modification of the *Bhlhe40* locus.

## Discussion

In this study, we show that the pathogenic effector program, but not the differentiation and non-pathogenic functions of tissue Th17 cells, is specifically controlled by the genome organizer Satb1. Satb1-mediated regulation of Bhlhe40 and PD-1 controls Th17 pathogenicity in part by IL-17 and GM-CSF production in the central nervous system during autoimmune disease. Notably, Satb1 differentially controlled the gene expression profiles in non-pathogenic and pathogenic Th17 cells isolated from the gut and inflamed tissue, respectively. Therefore, Satb1 shaped the divergent gene expression in tissue Th17 cells in an environmental context-dependent manner.

Satb1 regulates a specific set of gene expression in effector Th17 cells under inflammatory conditions. It is well appreciated that Satb1 is regulated by TCR signaling and highly expressed by immature thymocytes and plays a crucial role under physiological conditions in the specification and function of T cell lineages, including Foxp3^+^ Tregs developed in the thymus, by regulating the chromatin-loop architecture^33, 34, 46, 49^. It is of note that the expression levels of Satb1 are greatly reduced in mature T cells populating the periphery, implying that peripheral T cell subsets may decrease the dependency of Satb1 on their functions, and, for instance, its expression in Tregs has little impact on their functions and the Treg-mediated immunological self-tolerance in the periphery^29, 32, 34, 39, 49–51^. Although the knockdown of Satb1 reduced IL-17 expression in in vitro-polarized Th17 cells^52^ and the overexpression of SATB1 was observed in patients with anaplastic T lymphoma and increased Th17-associated genes^53^, the role of Satb1 in the differentiation and function of effector Th17 cells in vivo in autoimmune reactions remained elusive. Our results indicate that Satb1 is dispensable for the differentiation and cytokine production of gut Th17 cells, but significantly contributes to inflammatory properties of tissue Th17 cells, consistent with transcriptomic analysis revealing that the absence of Satb1 expression did not affect the fundamental gene expression profile in Th17 cells, unlike immature T cells and its precursors, but specifically controlled a limited number of effector genes in pathogenic Th17 cells in inflamed tissues in EAE mice. Hence, in vivo phenotypes of Th17 cells, such as the activation status, migratory capacity, and maintenance of RORγt and IL-17 expression, were not significantly affected by Satb1 whereas among Bhlhe40, Blimp-1, GM-CSF, JunB, Gpr65, and Toso, as key pathogenic factors expressed by Th17 cells in EAE^18, 19, 27, 44, 45, 54, 55^, Satb1 specifically regulates GM-CSF in part via Bhlhe40 expression and down-regulates PD-1 expression for the acquisition of a pathogenic mode of Th17 cells.

The gene expression of Bhlhe40 and PD-1 in effector T cells is controlled upon T cell activation and external environmental signals. CD28 stimulation is known to induce Bhlhe40 expression in T cells and IL-1R signaling further enhances it in Th17 cells^44, 56^. Given that Satb1-deficient Th17 cells express normal levels of IL-1R, reduced expression of Bhlhe40 may be due to disruption of its transcriptional machinery mediated by a transcription factor complex with Satb1 downstream of IL-1R signaling pathway, resulting in impaired GM-CSF production by effector Th17 cells. Notably, the dysregulation of PD-1 by Satb1 was only observed in Th17 cells from the spinal cord in EAE mice, but not in those from the draining LNs and PPs. This Satb1-mediated regulation of PD-1 during inflammation is likely to be indirect since no direct binding of Satb1 to the *Pdcd1* promoter region was detected. Inhibition of PD-1 occurs in CD8^+^ T cells through the recruitment of a nucleosome remodeling deacetylase repression complex by Satb1 and is abrogated in tumor micro-environments^42^, suggesting that similar mechanisms may operate in Th17 cells in inflamed tissues of EAE. Because Satb1 alone is not sufficient to change the expression levels of PD-1, external environmental cues such as chronic IL-23 signaling in inflamed tissues may be necessary to not only up-regulate the expression of Satb1, but also to modulate a mode of Satb1 function and dynamic chromatin architecture, shifting Th17 cells toward a pathogenic profile. On the other hand, in vitro treatment of TGF-β increased *Pdcd1* expression in Th17 cells, suggesting that TGF-β enriched environment such as the intestine may maintain Satb1 expression low and in turn prevent the down-regulation of PD-1 in non-pathogenic Th17 cells (Supplementary Fig. 5). Further studies are necessary to investigate how particular environmental signals and specific co-factors dictate Satb1 function to regulate the effector profile in Th17 cells, and how Satb1-mediated chromatin loop organization occurs in gene loci related to Th17 cell pathogenicity.

Because Satb1 is a key regulator of Th17 pathogenicity and its GM-CSF production in autoimmune disease in mice, manipulating Satb1 gene expression or function may be a therapeutic target for various autoimmune diseases in light of accumulating evidence that GM-CSF-targeting treatments have been reported to be effective in clinical trials for patients with rheumatoid arthritis and multiple sclerosis^57–61^. Furthermore, a single nucleotide polymorphism (rs11719975) in a region near the human *SATB1* gene has been recently associated with multiple sclerosis, suggesting an unappreciated link between *SATB1* and the pathogenic function of effector T cells in the central nervous system of the patients^62^. However, the SATB1-mediated gene expression and effector program of human Th17 cells could differ in patients with different inflammatory diseases and at different clinical stages, and should carefully be assessed using clinical samples in the future study. Together, our findings, in addition to providing novel insights into the molecular mechanisms underlying the pathogenic program of tissue Th17 cells in mice, may help design novel immunotherapeutic approaches such as small molecule modifiers of Satb1 for the treatment of autoimmune diseases.

## Methods

### Mice

C57BL/6J mice were purchased from CLEA Japan. *Rag2*^−/−^ mice have been previously described^63^. To generate *Satb1* conditional knockout mice, we crossed *Satb1*^fl/fl^ mice with *Il17a*^Cre^ *R26R*^eYFP^ or *Thpok*^Cre^ mice, in which Satb1 is depleted in IL-17-producing T cells or peripheral CD4^+^ T cells, respectively^35, 39, 46, 56^. All the mice used were on a C57BL/6 background and were maintained under SPF conditions in the animal facility at the Institute for Frontier Life and Medical Sciences, Kyoto University. Six to twelve-week-old mice were used for most of the experiments. Four-week-old mice were used for the analysis of thymocytes. All the experiments were approved by the animal ethical committee of the Institute for Frontier Life and Medical Sciences, Kyoto University and were performed in accordance with institutional guidelines.

### Antibodies

The following antibodies were used for the flow cytometry analysis and cell sorting: CD4 (1:400, RM4-5, #553051), CD8 (1:400, 53-6.7, #553033), CD44 (1:400, IM7, #553134), CD25 (1:200, PC61, #562606), IL-2 (1:200, JES6-5H4, #554428), IL-4 (1:200, 11B11, #554435), IL-10 (1:200, JES5-16E3, #554467), IL-17 (1:200, TC11-18H10, #560184), GM-CSF (1:200, MP1-22E9, #564747), PD-1 (1:200, J43, #562584), CTLA-4 (1:200, UC10-4F10-11, #553720), and GITR (1:200, DTA-1, #558140) antibodies were purchased from BD Biosciences. Ki67 (1:100, anti-human, clone B56, #556027), CD103 (1:100, M290, #557495), and ICOS (1:200, 7E.17G9, #552146) antibodies were purchased from BD Pharmingen. IFN-γ (1:200, XMG1.2, #25-7311-41), Foxp3 (1:200, FJK-16s, #12-5773-82), LAG3 (1:200, C9B7W, #17-2231-82), Tim3 (1:200, RMT3-23, #12-5870-82), and KLRG1 (1:200, 2F1, #25-5893-82) antibodies were purchased from eBioscience. TIGIT (1:200, 1G9, #142103) and Bcl2 (1:100, BCL/10C4, #633503) antibodies were purchased from Biolegend. NGFR (1:800, NGFR5, #MS-394-B1) and Live/Dead cell stain kit (#L34955) were purchased from Thermo Fisher Scientific.

### Cell preparation

For the preparation of double-positive (DP), CD4^+^ single-positive (SP), and CD8^+^ SP thymocytes, CD4^+^ CD8^+^, CD4^+^ CD8^−^, and CD4^−^ CD8^+^ thymocytes, respectively, were sorted using FACSAria (BD Bioscience). Peripheral naive (CD44^low^ CD25^−^ CD4^+^) T cells, regulatory (CD25^high^ CD4^+^) T cells and effector (CD44^high^ CD25^−^ CD4^+^) T cells were sorted from LNs and the spleen. CD4^+^ eYFP^+^ T cells from the PPs of non-immunized mice or the spinal cord of EAE mice at the peak of the disease (14 ± 3 days after EAE induction) were sorted by FACSAria as non-pathogenic or pathogenic Th17 cells, respectively. CD4^+^ eYFP^+^ T cells from draining LNs were sorted on day 7 after EAE induction. The pathogenic Th17 cells were prepared by mashing the spinal cord through a 70-μm mesh filter, followed by 36.5% Percoll separation.

### Cell culture

Purified CD25^−^ CD44^low^ CD4^+^ naive T cells were stimulated with plate-bound anti-CD3 (1 μg/ml; 2C11; BD Biosciences) and anti-CD28 (5 μg/ml; 37.51; BD Biosciences) in IMDM supplemented with 10% FCS, 100 U/ml penicillin, 100 μg/ml streptomycin, 50 μM 2-mercaptoethanol and 2 mM L-glutamine in the presence of rmIL-12 (10 ng/ml, R&D Systems) for Th1, rmIL-4 (10 ng/ml, R&D Systems) and anti-IFNγ (10 μg/ml, Biolegend) for Th2, rhTGF-β (10 ng/ml, R&D Systems) for iTregs, and rhTGF-β (1 ng/ml), rmIL-6 (20 ng/ml, R&D Systems) and rmIL-1β (20 ng/ml, R&D Systems) for Th17 conditions and intracellular cytokine and Foxp3 staining was performed after 72–96 h. In brief, for the cytokine staining, cells were restimulated for 2.5 h with PMA (500 ng/ml) and ionomycin (500 ng/ml) in the presence of brefeldin A (1 μg/ml). Fixation and permeabilization were followed by intracellular staining according to the manufacturer’s instruction (Thermo Fisher). Purified eYFP^+^ Th17 cells (1 × 10^6^/ml) from the draining LNs of the EAE mice were stimulated with CD3/CD28 Dynabeads (Thermo Fisher) in the presence or absence of rmIL-1β (20 ng/ml, R&D Systems), rmIL-6 (20 ng/ml, R&D Systems), rmIL-23 (100 ng/ml, R&D Systems) or rhTGF-β (10 ng/ml) for 24 h, and the cells were harvested for quantitative RT-PCR. Purified eYFP^+^ Th17 cells (5 × 10^4^/ml) from the spinal cord of EAE mice were stimulated with plate-bound anti-CD3 (1 μg/ml) or BMDCs (5 × 10^4^/ml) in the presence of MOG_35–55_ peptide (50 μg/ml) and anti-PD-1 mAb (20 μg/ml; 29F.1A12 from Bio X cell) or an isotype-matched control IgG2a (20 μg/ml; C1.18.4 from Bio X cell) for 24 h and cytokine concentrations in the supernatants were determined using a Cytometric Bead Array (BD Biosciences).

### Generation of bone marrow-derived dendritic cells (BMDCs)

Single cell suspensions of bone marrow cells from 4–8-week old C57BL/6J mice were prepared from the femur and tibia and subjected to red blood cell lysis with buffered ammonium chloride solution. Cells were then cultured at 1 × 10^6^/ml in RPMI supplemented with 10% fetal calf serum, penicillin, streptomycin, and 2-ME in the presence of GM-CSF (20 ng/ml) (R&D Systems). All media was removed and replaced with fresh media containing GM-CSF on day 3 and day 5. By day 7, more than 80% of the resulting cells expressed CD11b and class II MHC and were used as antigen presenting cells.

### EAE

In brief, mice were injected subcutaneously at two sites with an emulsion of 100 μl CFA and 250 μg MOG peptide (amino acids 35–55). Mice received 200 ng *Bordetella pertussis* (Calbiochem) intraperitoneally at day 0 and day 2 after EAE induction. An anti-PD-1 mAb (200 μg/mouse; 29F.1A12 from Bio X Cell) or a control IgG2a (200 μg/mouse; C1.18.4 from Bio X Cell) antibody was intraperitoneally injected three times a week beginning at day 5 after EAE induction. The clinical scores were assessed daily as follows: 0, unaffected; 1, flaccid tail; 2, impaired righting reflex and/or gait; 3, partial hind limb paralysis; 4, total hind limb paralysis; and 5, total hind limb paralysis with partial forelimb paralysis.

### Quantitative RT-PCR

Total RNA was extracted and reverse-transcribed using TRIzol Reagent (Life Technologies) and SuperScript VILO (Invitrogen) according to the manufacturer’s instruction. Quantitative RT-PCR was performed using TaqMan Gene Expression Assays (Thermo Fisher) and a Light cycler 480 (Roche) for the following genes: *Bhlhe40* (Mm00478593_m1), *Csf2* (Mm01290062_m1), *Foxp3* (Mm00475162_m1), *Hif1a* (Mm00468869_m1), *Ikzf3* (Mm01306721_m1), *Ifnγ* (Mm01168134_m1), *Il4* (Mm00445259_m1), *Il17a* (Mm00439618_m1), *Il22* (Mm00444241_m1), *Il23r* (Mm00519943_m1), *Nfkbiz* (Mm00600522_m1), *Pdcd1* (Mm01285676_m1), *Prdm1* (Mm00476128_m1), *Rorc* (Mm01261022_m1), *Satb1* (Mm01268937_m1), *Tbx21* (Mm00450960_m1), and *Hprt* (Mm03024075_m1), as a housekeeping gene. The target gene expression was calculated by the comparative method for relative quantification after normalization to Hprt expression.

### Cloning of *Bhlhe40* and retroviral transduction

Murine Bhlhe40 was amplified using 5′-TACGTAATGGAACGGATCCCCAGCGC-3′ and 5′-GTCGACTTAGTCTTTGGTTTCTACGT-3′ oligos and was cloned into the retroviral GCDN samI/N vector containing NGFR. The generation of retrovirus and the subsequent retroviral transduction were performed. In brief, retroviral plasmids were transfected into Plat-E cells using Fugene 6 (Promega) according to the manufacturer’s instructions and retrovirus-containing supernatants were harvested 48 h after transfection. Magnetically sorted CD4^+^ T cells were activated with plate-coated anti-CD3/CD28 for day 3 and the cells were spin-infected with retroviral supernatants containing 2.5 μg/ml polybrene for 90 min at 32 °C in 24-well plates at 1220*g*. The transduction efficiencies were assessed for NGFR expression, and the NGFR^+^ CD4^+^ T cells were sorted on day 5 after transduction.

### ChIP-sequencing

A total of 1–2.5 × 10^6^ Th0 or eYFP^+^ Th17 cells were induced in vitro under Th0 or Th17 culture conditions, and the cells were sorted for ChIP-seq analysis^34^. In brief, sorted cells were cross-linked using 1% formaldehyde and lysed. Cross-linked DNA was fragmented by sonication and the lysate was incubated with anti-H3K27ac (GeneTex, GEX60815) or anti-Satb1 (Abcam, ab70004) antibodies (Thermo Fisher) captured by magnetic beads, followed by elution, reverse cross-link, and purification for ChIP-seq analysis according to the manufacturer’s instructions. The ChIP-seq reads were mapped to the mouse genome mm9 Illumina iGenomes (http://support.illumina.com/sequencing/sequencing_software/igenome.html) using Bowtie2 (version 2.2.1), and the ChIP-seq peaks, normalized by total mapped read counts, were visualized in Integrative Genomics Viewer (Broad Institute).

### Quantitative ChIP-PCR

A total of 1–2.5 × 10^6^ Th0 or eYFP^+^ Th17 cells were induced in vitro under Th0 or Th17 culture conditions, and the cells were sorted for ChIP-qPCR. ChIP-qPCR analysis was performed with anti-Satb1 (Abcam, ab70004) antibody and control IgG (Abcam, ab171870) and the primers specific for the active promoter region of *Bhlhe40* (5′-CAATGACGACTGACCCACCA-3′ and 5′-CCCTGCAAGTTCGGAGAGTT-3′).

### RNA-sequencing

A total of 2 × 10^4^ eYFP^+^ Th17 cells from the spinal cord of the control and Th17^*Satb1*KO^ EAE mice were sorted at the peak of the disease (14 ± 3 days after EAE induction) for RNA-seq analysis in duplicate. A total of 1 × 10^4^ eYFP^+^ Th17 cells from the control and Th17^*Satb1*KO^ PPs of non-immunized mice were sorted for RNA-seq analysis in quadruplicate. In brief, total RNA was extracted and reverse-transcribed using RNeasy Micro Kit (Qiagen) and RNA-seq was performed using a SMART-seq v4 Ultra Low Input RNA Kit for Sequencing (Clonetech). The sequences were mapped to the mouse genome version mm9 using Hisat2. Differential gene expression analyses were performed using DESeq2 package in R (version 3.1.2) on tag counts obtained by HT-seq (version 0.6.1). The differentially expressed genes were defined with FDR less than 0.05.

### Statistical analysis

Two-tailed Student’s *t*-test was used for most of the statistical analyses (GraphPad Prism), and a *P* value of <0.05 was considered statistically significant. The clinical scores of EAE between each group were analyzed by two-way ANOVA with Bonferroni’s post-test.

### Reporting summary

Further information on experimental design is available in the Nature Research Reporting Summary linked to this article.

## Supplementary information

Supplementary Information

Reporting Summary

## Data Availability

ChIP-seq and RNA-seq datasets are available under the accession numbers DRA006772 and DRA007314 in DNA Data Bank of Japan [http://ddbj.nig.ac.jp/DRASearch/]. All relevant data are available upon reasonable request.
