# Supplementary material for: Satb1 regulates the effector program of encephalitogenic tissue Th17 cells in chronic inflammation
Source: Nat Commun. 2019 Feb 1;10:549. doi: 10.1038/s41467-019-08404-w (PMC6358604; doi:10.1038/s41467-019-08404-w)
Supplement: Supplementary file 1 — Supplementary Information [file 41467_2019_8404_MOESM1_ESM.pdf]

SUPPLEMENTARY INFORMATION

Satb1 regulates the effector program of encephalitogenic tissue Th17 cells in chronic inflammation

Keiko Yasuda, Yohko Kitagawa, Ryoji Kawakami, Yoshitaka Isaka, Hitomi Watanabe, Gen Kondoh, Terumi Kohwi-Shigematsu, Shimon Sakaguchi, Keiji Hirota

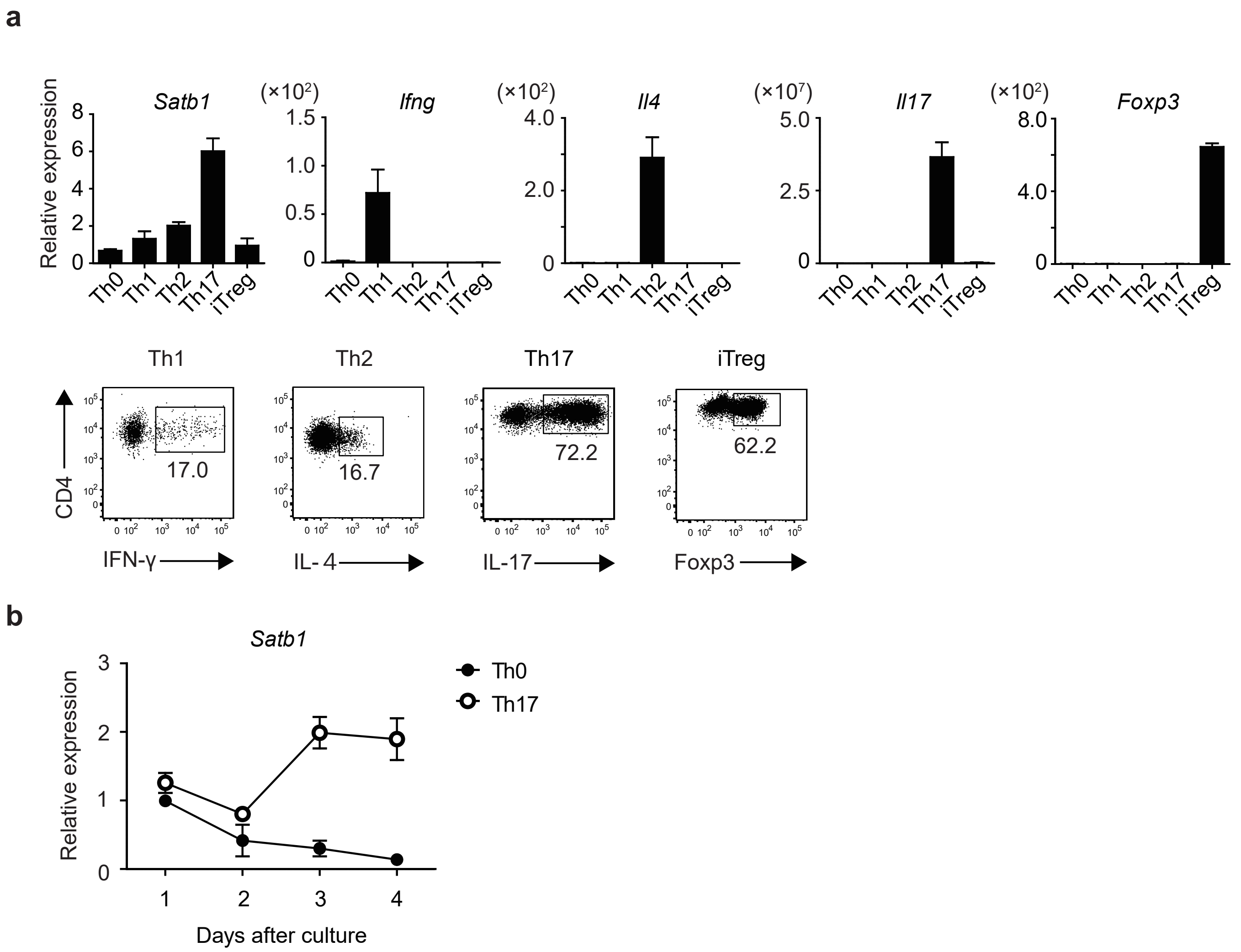

Supplementary Figure 1. Up-regulation of Satb1 mRNA under Th17 culture conditions. a) qPCR of Th0, Th1, Th2, Th17, and iTreg for *Satb1, Ifng, Il4, Il17a* and *Foxp3* mRNA. Flow cytometry of the indicated Th subsets and iTreg for intracellular IFN-γ, IL-4, IL-17 and Foxp3 expression. The bar graphs show the mean ± s.d. (n=3).
b) qPCR of Th0 and Th17 for the kinetics of *Satb1* mRNA. The results are representative of three independent experiments.

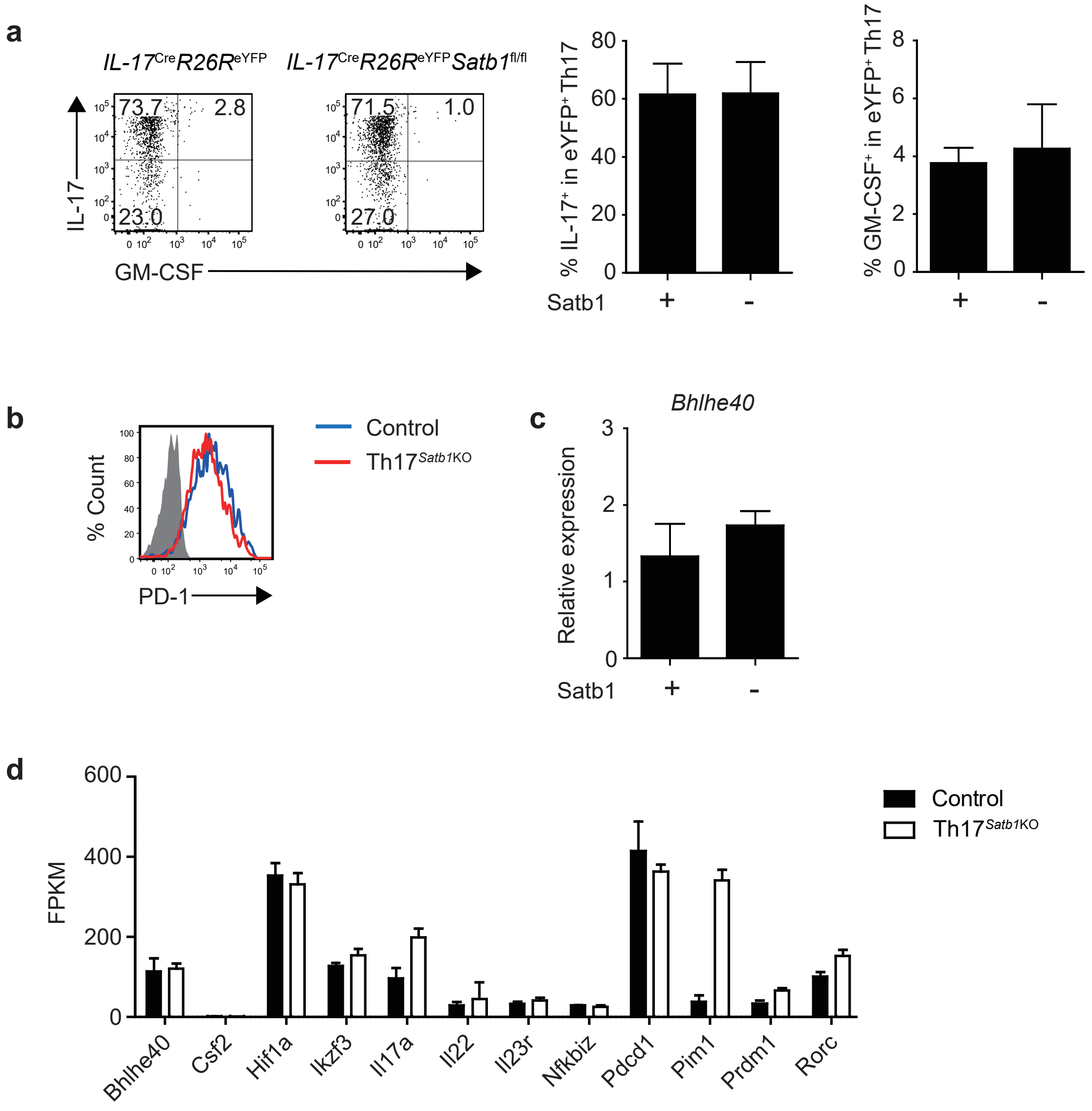

Supplementary Figure 2. Normal phenotypes and functions of Satb1-deficient non-pathogenic Th17 cells in Peyer' s patches. a) Flow cytometry of the PPs eYFP+ CD4+ T cells from *Il17a*^{Cre} *R26R*^{eYFP} *Satb1*$^{wt/wt}$ (control) and *Il17a*^{Cre} *R26R*^{eYFP} *Satb1*$^{fl/fl}$ (Th17^{Satb1KO} mice) for intracellular IL-17A and GM-CSF expression. The frequencies of IL-17+ and GM-CSF+ in eYFP+ CD4+ T cells are shown. **b)** Flow cytometry of the PPs eYFP+ CD4+ T cells as in (**a**) for PD-1 expression. **c)** qPCR of the expression of *Bhlhe40* in the PPs eYFP+ CD4+ T cells of control or Th17^{Satb1KO} mice. The bar graphs (**a** and **c**) show the mean ± s.d. (n=3). The results are representative of at least three independent experiments (**a-c**). **d)** RNA-seq analysis of the PPs eYFP+ CD4+ T cells from control and Th17^{Satb1KO} mice. The data are presented as fragments per kilo base of exon per million mapped fragments (FPKM). The bar graphs show the mean ± s.d. (WT control; n=3, Satb1 CKO; n=4).

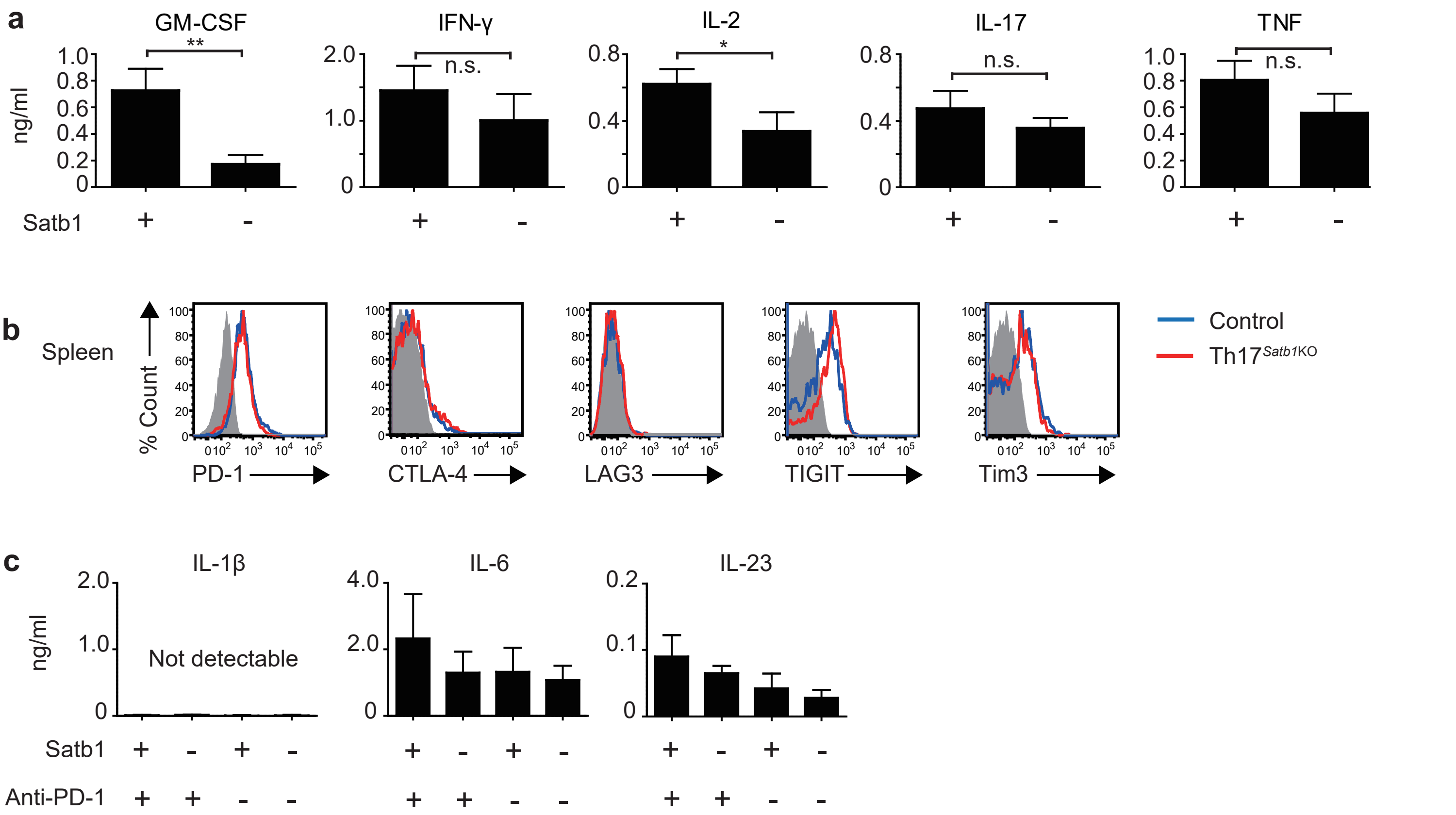

Supplementary Figure 3. Cytokine production and the expression of surface markers by Th17 cells.

a) Cytokine concentrations in the culture supernatant of re-stimulated Th17 cells are shown. $eYFP^+$ Th17 cells were sorted from the spleen of control or Th17^{Satb1KO} mice at the peak of EAE. Sorted Th17 cells were re-stimulated with BMDCs in the presence of $MOG_{35\text{-}55}$ peptide (50 μg/ml) for 24 hours. **b)** Flow cytometry of splenic $eYFP^+$ $CD4^+$ T cells (day14 after EAE induction) for the expression of PD-1, CTLA-4, LAG3, TIGIT, and Tim3. **c)** Cytokine concentrations in the culture supernatant of re-stimulated Th17 cells are shown. $eYFP^+$ Th17 cells were sorted from the spinal cord of control or Th17^{Satb1KO} mice at the peak of EAE. Sorted Th17 cells were re-stimulated with BMDCs in the presence of $MOG_{35\text{-}55}$ peptide (50 μg/ml) with or without the PD-1 antibody (20 μg/ml) for 24 hours. The bar graphs (**a** and **c**) show the mean ± s.d. (n=3). *P<0.05; **P<0.001. (two-tailed unpaired Student’ s *t*-test)

a

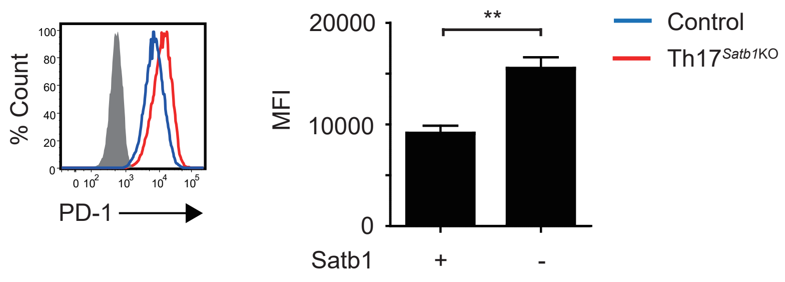

b

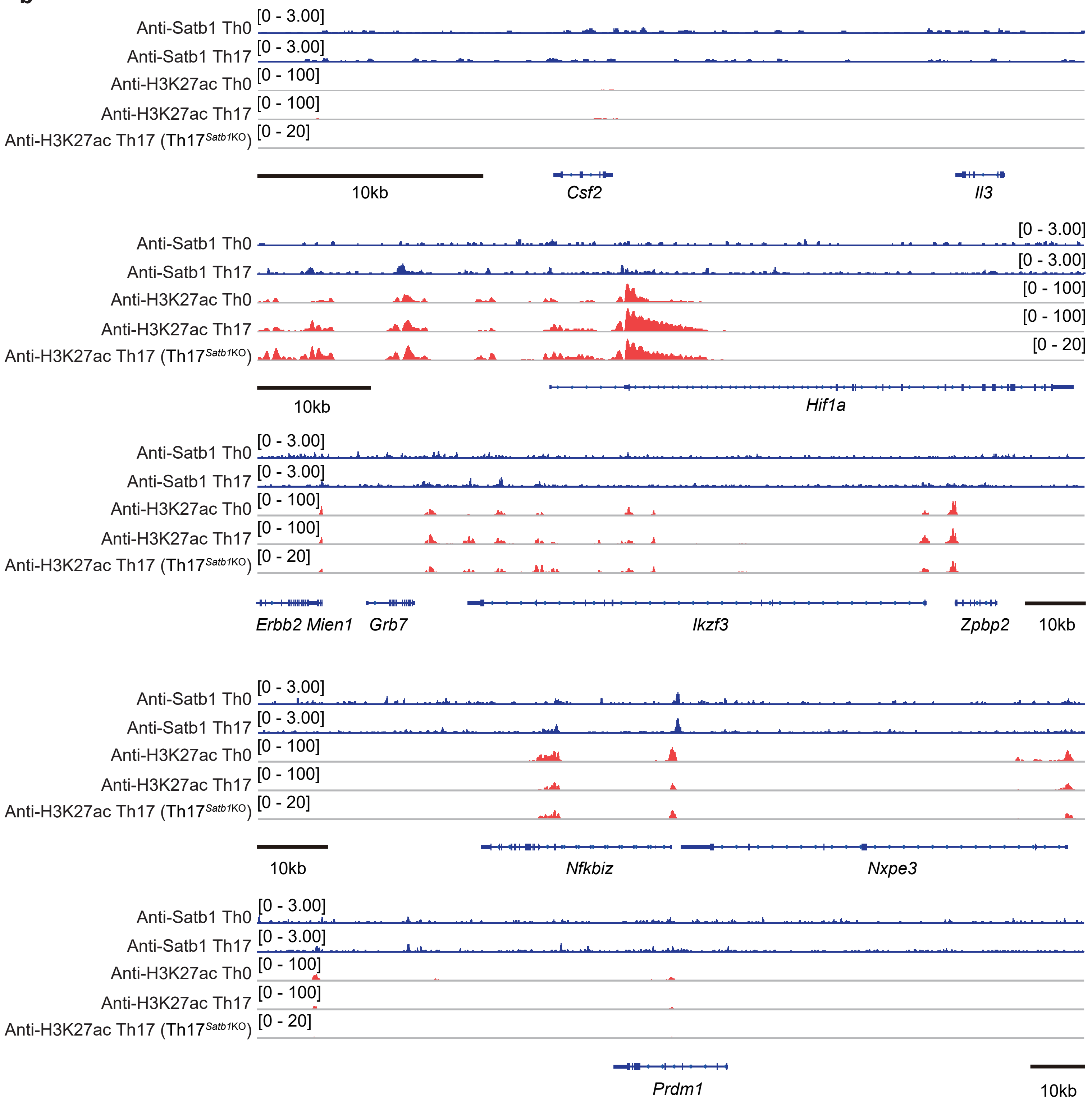

Supplementary Figure 4

Supplementary Figure 4. Analysis of the Satb1 binding sites at the *Csf2, Hif1a, Ikzf3, Nfkbiz* and *Prdm1* loci. **a)** Flow cytometry of *in vitro* polarized Th17 cells from control and Th17^{Satb1KO} mice for PD-1 expression. The bar graphs show the mean ± s.d. (n=3). **P<0.001(two-tailed unpaired Student' s *t*-test). **b)** ChIP-seq analyses of Satb1 binding and H3K27ac modification at the *Csf2, Hif1a, Ikzf3, Nfkbiz* and *Prdm1* loci in *in vitro* polarized Th0 or eYFP^{+} Th17 cells.

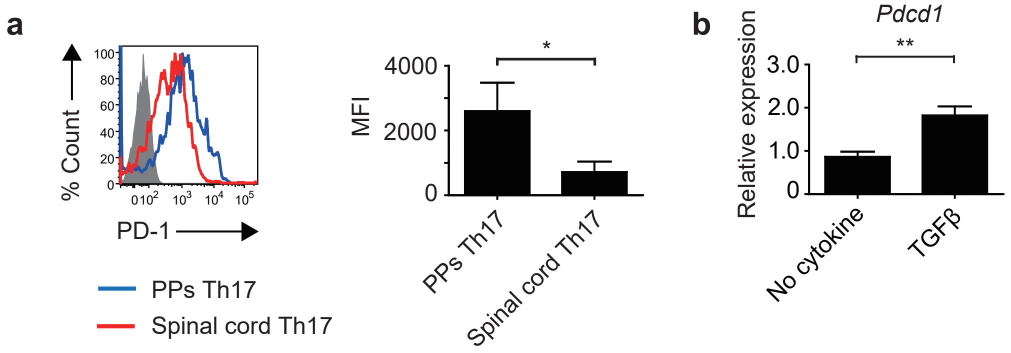

Supplementary Figure 5. PD-1 expression by Th17 cells from PPs and inflamed spinal cord.

a) Flow cytometry of eYFP$^+$ CD4$^+$ T cells from PPs and the spinal cord 14 days after EAE induction for the expression of PD-1. **b)** qPCR of *Pdcd1* mRNA expression in re-stimulated Th17 cells are shown. eYFP$^+$ Th17 cells were sorted from the draining LNs of EAE mice on day7 and then re-stimulated with CD3/CD28 Dynabeads in the presence TGFβ for 24 hours. The bar graphs (**a** and **b**) show the mean ± s.d. (n=3). The results are representative of three independent experiments. **P*<0.05; ***P*<0.001. (two-tailed unpaired Student' s *t*-test).

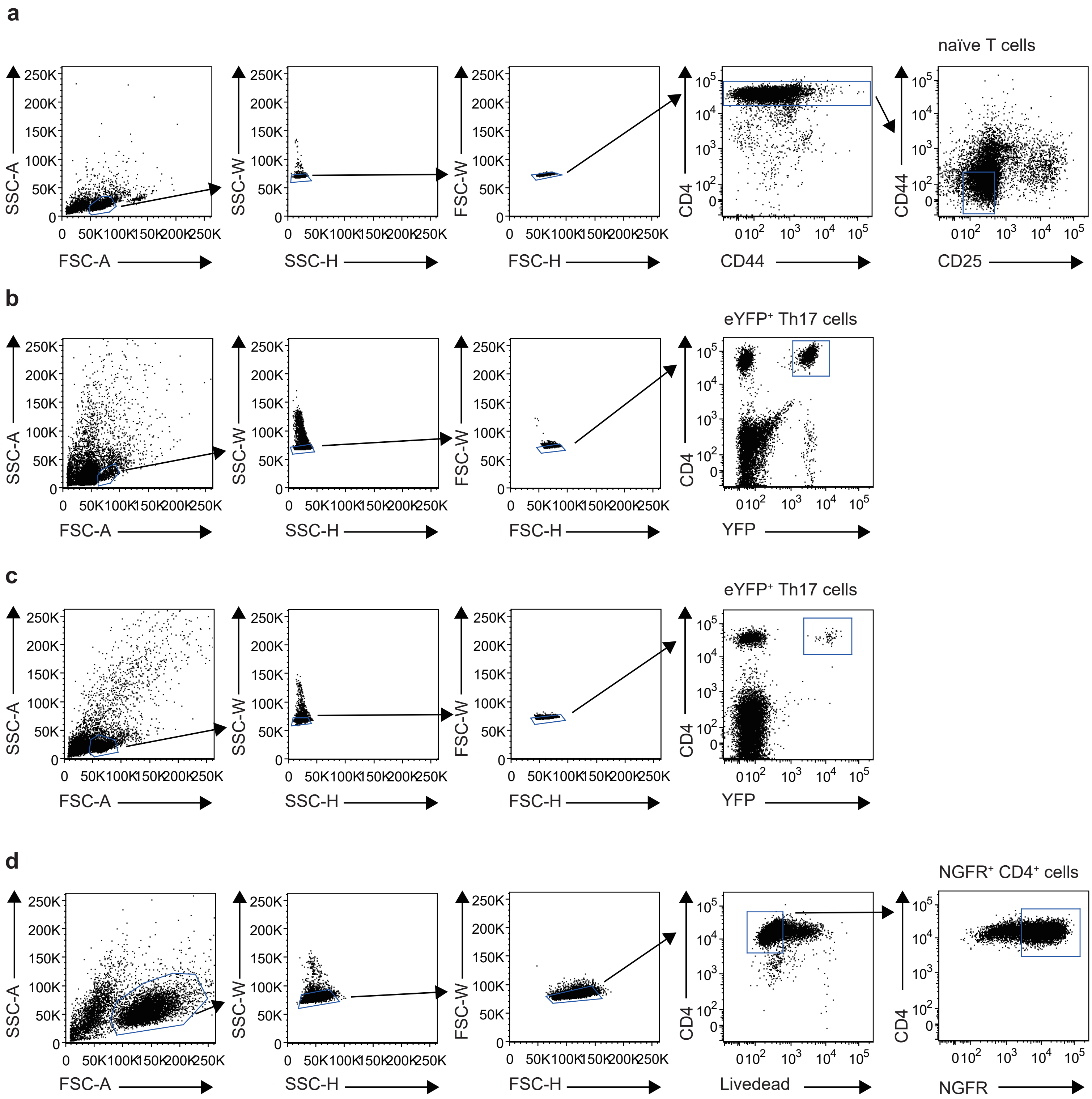

Supplementary Figure 6. Gating strategies. a) Gating strategies to sort naïve (CD44^{low} CD25$^-$ CD4$^+$) T cells from C57BL/6 WT mice presented on **Fig. 1a**. The same strategy was used to sort naïve T cells presented on **Fig. 1, 5, 7, Supplementary Fig. 1** and **Supplementary Fig 4**. **b)** Gating strategies to sort pathogenic eYFP$^+$ Th17 cells from the spinal cord after EAE induction from control mice presented on **Fig. 2g**. The same strategy was used for the analysis and sorting of pathogenic Th17 cells in **Fig. 2, 3, 4, 5, 6, Supplementary Fig. 3, Supplementary Fig. 5** and **Table 2**. **c)** Gating strategies for non-pathogenic eYFP$^+$ Th17 cells from the PPs of non-immunized control mice presented on **Fig.1a**. The same strategy was used for the analysis and sorting of non-pathogenic Th17 cells in **Fig. 2, 4, 5, Supplementary Fig. 2, Supplementary Fig. 5**, and **Table 2**. **d)** Gating strategies to sort NGFR$^+$ CD4$^+$ T cells after retroviral transduction presented on **Fig. 5c**. The same strategy was used for sorting of NGFR$^+$ CD4$^+$ T cells in **Fig. 5**.
