# Supplementary material for: Satb1 regulates the effector program of encephalitogenic tissue Th17 cells in chronic inflammation
Source: Nat Commun. 2019 Feb 1;10:549. doi: 10.1038/s41467-019-08404-w (PMC6358604; doi:10.1038/s41467-019-08404-w)
Supplement: Supplementary file 2 — Reporting Summary [file 41467_2019_8404_MOESM2_ESM.pdf]

nature research

Corresponding author(s): Keiji Hirota

Reporting Summary

Nature Research wishes to improve the reproducibility of the work that we publish. This form provides structure for consistency and transparency in reporting. For further information on Nature Research policies, see Authors & Referees and the Editorial Policy Checklist.

Statistical parameters

When statistical analyses are reported, confirm that the following items are present in the relevant location (e.g. figure legend, table legend, main text, or Methods section).

n/a	Confirmed	
☐	☒	The exact sample size (*n*) for each experimental group/condition, given as a discrete number and unit of measurement
☐	☒	An indication of whether measurements were taken from distinct samples or whether the same sample was measured repeatedly
☐	☒	The statistical test(s) used AND whether they are one- or two-sided<br>*Only common tests should be described solely by name; describe more complex techniques in the Methods section.*
☒	☐	A description of all covariates tested
☒	☐	A description of any assumptions or corrections, such as tests of normality and adjustment for multiple comparisons
☒	☐	A full description of the statistics including central tendency (e.g. means) or other basic estimates (e.g. regression coefficient) AND variation (e.g. standard deviation) or associated estimates of uncertainty (e.g. confidence intervals)
☐	☒	For null hypothesis testing, the test statistic (e.g. *F*, *t*, *r*) with confidence intervals, effect sizes, degrees of freedom and *P* value noted<br>*Give P values as exact values whenever suitable.*
☒	☐	For Bayesian analysis, information on the choice of priors and Markov chain Monte Carlo settings
☒	☐	For hierarchical and complex designs, identification of the appropriate level for tests and full reporting of outcomes
☒	☐	Estimates of effect sizes (e.g. Cohen's *d*, Pearson's *r*), indicating how they were calculated
☐	☒	Clearly defined error bars<br>*State explicitly what error bars represent (e.g. SD, SE, CI)*

Our web collection on statistics for biologists may be useful.

Software and code

Policy information about availability of computer code

Data collection	No software was used to the data collection.
Data analysis	Statistical analysis was performed using GraphPad Prism 5 (Graph Pad Software).

For manuscripts utilizing custom algorithms or software that are central to the research but not yet described in published literature, software must be made available to editors/reviewers upon request. We strongly encourage code deposition in a community repository (e.g. GitHub). See the Nature Research guidelines for submitting code & software for further information.

Data

Policy information about availability of data

All manuscripts must include a data availability statement. This statement should provide the following information, where applicable:

- Accession codes, unique identifiers, or web links for publicly available datasets
- A list of figures that have associated raw data
- A description of any restrictions on data availability

Accession codes; source data for figures are provided with the paper.

April 2018

Field-specific reporting

Please select the best fit for your research. If you are not sure, read the appropriate sections before making your selection.

- [x] Life sciences
- [ ] Behavioural & social sciences
- [ ] Ecological, evolutionary & environmental sciences

For a reference copy of the document with all sections, see nature.com/authors/policies/ReportingSummary-flat.pdf

Life sciences study design

All studies must disclose on these points even when the disclosure is negative.

Sample size	Animal sample size estimations were determined using previous studies using 3-6 animals per group and guided by the representative of at least three independent experiments.
Data exclusions	No data exclusions were performed.
Replication	All experimental investigations except ChIP-seq and RNA-seq analysis were reliably reproduced guided by the representative of more than three independent experiments. RNA-seq was carried on 2-4 biological replicates. The ChIP-seq data is the result of single experiment.
Randomization	Randomization was not carried out in this study. Animals were age and sex matched between experimental groups.
Blinding	Blinding was not performed during data collection. Blinding was performed during bioinformatic data analysis.

Reporting for specific materials, systems and methods

Materials & experimental systems

n/a	Involved in the study	
☒	☐	Unique biological materials
☐	☒	Antibodies
☒	☐	Eukaryotic cell lines
☒	☐	Palaeontology
☐	☒	Animals and other organisms
☒	☐	Human research participants

Methods

n/a	Involved in the study	
☐	☒	ChIP-seq
☐	☒	Flow cytometry
☒	☐	MRI-based neuroimaging

Antibodies

Antibodies used	The following antibodies were used for the flow cytometry analysis and cell sorting: CD4 (RM4-5), CD8 (53-6.7), CD44 (IM7), CD25 (PC61), IL-2 (JES6-5H4), IL-4 (11B11), IL-10 (JES5-16E3), IL-17 (TC11-18H10), GM-CSF (MP1-22E9), PD-1 (J43), CTLA-4 (UC10-4F10-11), and GITR (DTA-1) antibodies were purchased from BD Biosciences. Ki67 (anti-human, clone B56), CD103 (M290) and ICOS (7E.17G9) antibodies were purchased from BD Pharmingen. IFN-γ (XMG1.2), Foxp3 (FJK-16s), LAG3 (C9B7W), Tim3 (RMT3-23) and KLRG1 (2F1) antibodies were purchased from eBioscience. TIGIT (1G9) and Bcl2 (BCL/10C4) antibodies were purchased from Biolegend.NGFR (NGFR5) and Live/Dead cell stain kit was purchased from Thermo Fisher Scientific.
Validation	All the antibodies were validated against cells we used in this study.

Animals and other organisms

Policy information about studies involving animals; ARRIVE guidelines recommended for reporting animal research

Laboratory animals	C57BL/6J mice were purchased from CLEA Japan. Rag2-/- mice have been previously described. To generate Satb1 conditional knockout mice, we crossed Satb1 fl/fl mice with Il17aCre R26ReYFP or ThpokCre mice, in which Satb1 is depleted in IL-17-producing T cells or peripheral CD4+ T cells, respectively. All the mice used were on a C57BL/6 background and were maintained under SPF conditions in the animal facility at the Institute for Frontier Life and Medical Sciences, Kyoto University. Six to twelve-week-old mice were used for most of the experiments.
Wild animals	No wild animals were used in this study.
Field-collected samples	No field-collected samples were included in this study.

ChIP-seq

Data deposition

☒ Confirm that both raw and final processed data have been deposited in a public database such as GEO.

☐ Confirm that you have deposited or provided access to graph files (e.g. BED files) for the called peaks.

Data access links
May remain private before publication.

ChIP-seq and RNA-seq data sets were deposited in DNA Data Bank of Japan under the accession number DRA006772 and DRA007314.

Files in database submission

No	File_NAME_Curated	Application
1	01_Th17_spinal_cord_EAE_Control_01	RNA-seq
2	02_Th17_spinal_cord_EAE_Control_02	RNA-seq
3	03_Th17_spinal_cord_EAE_Satb1cKO_01	RNA-seq
4	04_Th17_spinal_cord_EAE_Satb1cKO_02	RNA-seq
5	05_Th17_PeyerPatch_Control_01	RNA-seq
6	06_Th17_PeyerPatch_Control_02	RNA-seq
7	07_Th17_PeyerPatch_Control_03	RNA-seq
8	08_Th17_PeyerPatch_Satb1cKO_01	RNA-seq
9	09_Th17_PeyerPatch_Satb1cKO_02	RNA-seq
10	10_Th17_PeyerPatch_Satb1cKO_03	RNA-seq
11	11_Th17_PeyerPatch_Satb1cKO_04	RNA-seq
12	21_Th0_H3K27ac_ChIPseq	ChIP-seq
13	22_Th17_H3K27ac_ChIPseq	ChIP-seq
14	23_Th0_Satb1_ChIPseq	ChIP-seq
15	24_Th17_Satb1_ChIPseq	ChIP-seq
16	25_Th0_H3K27ac_ChIPseq_rep2	ChIP-seq
17	26_Th17_H3K27ac_ChIPseq_rep2	ChIP-seq
18	27_Th17_H3K27ac_Satb1KO_rep1	ChIP-seq
19	28_Th17_H3K27ac_Satb1KO_rep2	ChIP-seq
20	29_Th0_Satb1_ChIPseq_rep2	ChIP-seq
21	30_Th17_Satb1_ChIPseq_rep2	ChIP-seq

Genome browser session
(e.g. UCSC)

Not available.

Methodology

Replicates

RNA-seq was carried on 2-4 biological replicates. The ChIP-seq data is the result of two experiments.
ChIP-qPCR was carried three biological replicates.

Sequencing depth

H3K27ac Th0 (21_Th0_H3K27ac_ChIPseq.fastq)
Total number of reads: 33391135
Uniquely mapped reads: 22351790
Length of reads: 150bp
Single-end
H3K27ac Th17 (22_Th17_H3K27ac_ChIPseq.fastq)
Total number of reads: 24760710
Uniquely mapped reads: 16473877
Length of reads: 150bp
Single-end
Satb1 Th0 (23_Th0_Satb1_ChIPseq.fastq)
Total number of reads: 9335214
Uniquely mapped reads: 6141512
Length of reads: 150bp
Single-end
Satb1 Th17 (24_Th17_Satb1_ChIPseq.fastq)
Total number of reads: 12672641
Uniquely mapped reads: 8631710
Length of reads: 150bp
Single-end
H3K27ac Th0 replicate (25_Th0_H3K27ac_ChIPseq_rep2)
Total number of reads: 10663823
Uniquely mapped reads: 7433976
Length of reads: 150bp
Single-end
H3K27ac Th17 replicate (26_Th17_H3K27ac_ChIPseq_rep2)
Total number of reads: 12000613
Uniquely mapped reads: 8322026
Length of reads: 150bp
Single-end
H3K27ac Th17 (Satb1cKO) (27_Th17_H3K27ac_Satb1KO_rep1)
Total number of reads: 12950990

	Uniquely mapped reads: 9133735<br>Length of reads: 150bp<br>Single-end<br>H3K27ac Th17 (Satb1cKO) replicate (28_Th17_H3K27ac_Satb1KO_rep2)<br>Total number of reads: 13663688<br>Uniquely mapped reads: 9543041<br>Length of reads: 150bp<br>Single-end<br>Satb1 Th0 replicate(29_Th0_Satb1_ChIPseq_rep2)<br>Total number of reads: 9371380<br>Uniquely mapped reads: 5157725<br>Length of reads: 150bp<br>Single-end<br>Satb1 Th17 replicate (30_Th17_Satb1_ChIPseq_rep2)<br>Total number of reads: 11786334<br>Uniquely mapped reads: 6389554<br>Length of reads: 150bp<br>Single-end
Antibodies	Anti-H3K27ac (GeneTex, GEX60815) and anti-Satb1 (Abcam, ab70004) antibodies were used.<br>For ChIP-qPCR, anti-Satb1 (Abcam, ab70004) antibodies and control IgG (Abcam, ab171870) were used.
Peak calling parameters	Reads were mapped using bowtie2 with mm9 index files.<br>Peaks were called using macs2 with default parameters. Peak calling was carried out by comparing ChIP DNA and input (DRP003376, Kitagawa et al. Nat Immunol 2017), which is fixed and fragmented DNA before immunoprecipitation.
Data quality	Peaks were visually analyzed by using IGV and following number of peaks were identified using macs2;<br>H3K27ac Th0: 19612<br>H3K27ac Th17: 20949<br>H3K27ac Th17 (Satb1cKO) :16766<br>Satb1 Th0: 1407<br>Satb1 Th17: 6375
Software	The ChIP-seq reads were mapped to the mouse genome mm9 illumina iGenomes (http://support.illumina.com/sequencing/sequencing_software/igenome.html) using Bowtie2 (version 2.2.1), and the ChIP-seq peaks, normalized by total mapped read counts, were visualized in Integrative Genomics Viewer (Broad Institute).

Flow Cytometry

Plots

Confirm that:

- [ ] The axis labels state the marker and fluorochrome used (e.g. CD4-FITC).
- [x] The axis scales are clearly visible. Include numbers along axes only for bottom left plot of group (a 'group' is an analysis of identical markers).
- [ ] All plots are contour plots with outliers or pseudocolor plots.
- [x] A numerical value for number of cells or percentage (with statistics) is provided.

Methodology

Sample preparation	CD4+ eYFP+ T cells from the PPs of non-immunized mice or the spinal cord of EAE mice at the peak of the disease (14±3 days after EAE induction) were sorted by FACSAria (BD Biosciences) as non-pathogenic or pathogenic Th17 cells, respectively. The pathogenic Th17 cells were prepared by mashing the spinal cord through a 70-µm mesh filter, followed by 36.5% Percoll separation.
Instrument	Sample to be analysed were acquired on BD FACS Aria II (Beckton Dickinson) cytometer. CD4+CD8+, CD4+, CD8+ cells from the thymus, CD8+, CD25-CD44lowCD4+, CD25-CD44highCD4+ and CD4+eYFP+ T cells were sorted on BD FACS Aria II (Beckton Dickinson) cytometer.
Software	Data was collected using Diva Softwares. All data were analysed using Flow Jo Software (Treestar).
Cell population abundance	CD25-CD44lowCD4+ naive T cells were sorted over 95% purity using a 4 way purity mode setting. Other population was also sorted over 95% purity.
Gating strategy	(Figure 1a, 1b)<br>For sorting or analysis of cells in thymus, cells were gated on lymphocytes in FSC/SSC gate, followed by exclusion of doublets in both FSC-A/FSC-H and SSC-A/SSC-H parameters, followed by gating on CD4+ CD8+, CD4+ CD8- or CD4-CD8+.<br><br>(Figure 1a, 5a)<br>For sorting of CD8+ Tcells, CD25-CD44lowCD4+ naïve T cells, CD25highCD4+ regulatory T cells, CD25-CD44highCD4+ effector/memory T cells were gated on lymphocytes in FSC/SSC gate, followed by exclusion of doublets in both FSC-A/FSC-H and SSC-A/

SSC-H parameters, followed by gating on CD8+ (CD8+ T cells) or gating on CD4+, followed by CD25-CD44low(naïve T cells) or CD25high(regulatory T cells) or CD25-CD44high(effector/memory T cells).

(Figure 1c, 1e, 1f, 2c, 2d, 2e, Supplementary Figure 1a)
For analysis of intracellular cytokines, cells were gated on live CD4+ T cells as follows: lymphocytes were gated in FSC/SSC gate, followed by exclusion of doublets in both FSC-A/FSC-H and SSC-A/SSC-H parameters, followed by gating on CD4+.

(Figure 1d)
For analysis of intracellular cytokines of transferred naïve T cells, cells were gated on live CD4+ T cells as follows: lymphocytes were gated in FSC/SSC gate, followed by exclusion of doublets in both FSC-A/FSC-H and SSC-A/SSC-H parameters, followed by gating on CD4+TCRβ+.

(Figure2f, 2g, 2h, 2i, 4b, 4c, 4d, 5a, 6a, 6e, Supplementary Figure 2a, 2d, 3a, 5)
For analysis of intracellular cytokines of Th17 cells, lymphocytes were gated in FSC/SSC gate, followed by exclusion of doublets in both FSC-A/FSC-H and SSC-A/SSC-H parameters, followed by gating on CD4+, followed by eYFP+

(Figure3a, 3b, 3c, 6b, 6c, 7a, Supplementary Figure 2b, 2c, 3b, 4a, 5a)
For analysis of protein expression in Th17 cells, lymphocytes were gated in FSC/SSC gate, followed by exclusion of doublets in both FSC-A/FSC-H and SSC-A/SSC-H parameters, followed by gating on CD4+, followed by eYFP+.

(Figure 5d, 5e)
For the analysis of intracellular cytokines of retrovilally transducted CD4+ T cells, cells were gated on live transducted CD4+ T cells as follows: lymphocytes were gated in FSC/SSC gate, followed by exclusion of doublets in both FSC-A/FSC-H and SSC-A/SSC-H parameters, followed by gating on live CD4+ T cells as defined by lack of dead cell dye in LIVE/DEAD Fixable Violet, followed by NGFR+.

☒ Tick this box to confirm that a figure exemplifying the gating strategy is provided in the Supplementary Information.
